# Influence of *Pleurotus sapidus* fruiting bodies on the performance, cecal microbiome, and gene expression in the liver and breast muscle of broilers

**DOI:** 10.1016/j.psj.2025.105517

**Published:** 2025-07-03

**Authors:** Lea Schäfer, Sarah M. Grundmann, Verena Hepp, Javier Herrero-Encinas, Martin Rühl, Erika Most, Robert Ringseis, Klaus Eder

**Affiliations:** a Institute of Animal Nutrition and Nutrition Physiology, Justus Liebig University Giessen, Giessen, Germany; b ETS Ingeniería Agronómica, Alimentaria y de Biosistemas, Departamento de Producción Agraria, Universidad Politécnica de Madrid, Madrid, Spain; c Institute of Food Chemistry and Food Biotechnology, Justus Liebig University Giessen, Giessen, Germany; d Fraunhofer Institute for Molecular Biology and Applied Ecology IME, Giessen, Germany; e Center for Sustainable Food Systems, Justus Liebig University Giessen, Giessen, Germany

**Keywords:** Broilers, Fungi, Gut microbiota, Liver transcriptome, Muscle protein turnover

## Abstract

Mushrooms, the fruiting bodies of edible fungi, are widely used as food for humans. However, their potential, as well as that of fungal mycelia, as feed components for poultry is less acknowledged. Recent studies have shown that feeding the vegetative mycelium of *Pleurotus sapidus* does not affect growth performance or nutrient digestibility and causes only minimal changes in the cecal microbiota structure, liver transcriptome, and plasma metabolome of broilers. The present study aimed to comprehensively investigate the effects of feeding the fruiting bodies of *P. sapidus* on performance metrics, ileal nutrient digestibility, cecal microbiota composition, cecal integrity, liver transcriptome, and the expression of genes involved in protein turnover in breast muscle of broilers. A total of 72 male, 1-day-old Cobb 500 broilers were randomly assigned to three groups and fed three distinct diets containing either 0 g (PSA-F0), 25 g (PSA-F25), or 50 g (PSA-F50) of freeze-dried *P. sapidus* fruiting bodies per kg diet in a 35-day, three-phase feeding regimen. Final body weights and weight gain during the finisher and the whole period were significantly lower in groups PSA-F50 and PSA-F25 compared to group PSA-F0 (*P* < 0.05). Feed intake during the finisher and the whole period tended to be lower in groups PSA-F50 and PSA-F25 compared to group PSA-F0 (*P* < 0.1). Average daily apparently digested amounts of most indispensable amino acids were lower in group PSA-F50 than in group PSA-F0 (*P* < 0.05). Cecal microbial α-diversity indicators (Chao1 and Richness) were significantly higher in the PSA-F50 group compared to the PSA-F0 group (*P* < 0.05), whereas β-diversity indicators were similar between groups. Taxonomic analysis showed a higher abundance of the class Bacilli and the species *unknown_Erysipelatoclostridium* and a lower abundance of the class Clostridia in the PSA-F50 group compared to the PSA-F0 group (*P* < 0.05). Concentrations of total and individual short-chain fatty acids, including acetic acid and propionic acid, in the cecal digesta were lower in the PSA-F50 group compared to the PSA-F0 group (*P* < 0.05). A total of 66 differentially expressed transcripts were identified in the liver between PSA-F50 and PSA-F0 groups based on filter criteria (FC > 1.3 or FC < -1.3, *P* < 0.05). The mRNA levels of genes involved in critical pathways such as protein synthesis and degradation—including the mammalian target of rapamycin pathway, myogenesis, the ubiquitin-proteasome system, autophagy-lysosomal pathway, and GCN2/eIF2α pathway—did not vary across the groups. Plasma lipopolysaccharide concentration was similar across all groups. The mRNA levels of *CLDN3, MUC2*, and *MUC5AC* were elevated in the PSA-F50 group compared to the PSA-F0 group (*P* < 0.05), while mRNA levels of *CLDN5, OCLN, MUC13*, and several pro-inflammatory genes in cecal mucosa remained unchanged across groups. The observed impairment in growth performance suggests that *P. sapidus* fruiting bodies cannot be recommended as dietary components for broilers at the tested doses. Considering the higher β-glucan content of fruiting bodies compared to vegetative mycelia, the negative effects observed on broiler performance may be associated with their β-glucan content.

## Introduction

The provision of sufficient amounts of adequate feed sources for farm animals represents a growing global challenge (Wu et al., 2014). While global meat production is expected to rise by more than 10 % in the next decade, with poultry meat accounting for 50 % of the additional production (OECD/FAO, 2024), the scarcity of natural resources continues to increase due to climate change, land degradation, water shortages, food-feed competition, and other factors. In light of this, it is becoming increasingly important to provide poultry feed in a sustainable, environmentally friendly, and resource-efficient manner.

Biomass derived from cultivated edible fungi, commonly referred to as mushrooms—such as *Pleurotus spp., Agaricus bisporus*, and *Lentinula edodes*—might be a suitable feed for broilers. These fungi are capable of efficiently converting low-value lignocellulosic side streams from agri-industrial and wood processing into high-value mycelial biomass (Ahlborn et al., 2019; Meyer et al., 2020), which can be used as feed. In addition to providing certain amounts of protein (Ayimbila and Keawsompong, 2023), biomass from these cultivated fungi is noteworthy for its rich composition of bioactive compounds, such as fermentable carbohydrates, phenolic compounds, and various pharmacologically active substances (e.g., ergothioneine, cordycepin, lovastatin, γ-aminobutyric acid) (Araújo-Rodrigues et al., 2024; Tsiantas et al., 2021). While edible mushrooms have a long-standing history as a nutritious and flavorful food for humans (Valverde et al., 2015; Muszyńska et al., 2018), their potential—as fruiting bodies, fungal mycelia, and by-products of mushroom production (such as stem residues and spent mushroom substrate)—as a feed component for poultry remains underexplored. Given the limited knowledge about fungal biomass as a poultry feed, we have recently investigated the effect of feeding two different doses (25 and 50 g/kg diet) of vegetative mycelium from *P. sapidus*, produced *via* submerged fermentation, on growth performance, cecal microbiota structure, gut integrity, nutrient digestibility, liver transcriptome, and plasma metabolome in broilers (Schäfer et al., 2024a). This study revealed that feeding *P. sapidus* mycelium neither impacts growth performance nor nutrient digestibility, while exerting only minimal effects on the cecal microbiota structure, liver transcriptome, and plasma metabolome of broilers. Furthermore, a complementary study from the same trial indicated no effect of feeding *P. sapidus* mycelium on the expression of genes related to protein turnover in breast muscle (Schäfer et al., 2024b).

Although the nutritional composition of vegetative mycelium and fruiting bodies is similar, significant differences exist in their β-glucan content, which is typically higher in fruiting bodies than in vegetative mycelium of cultivated fungi (Nitschke et al., 2011). The β-glucans, together with chitin, primarily function to stabilize the fungal cell wall and are well-documented fermentation substrates for gut microbes. These compounds modulate gut microbiota composition, influence gut integrity and function, and even impact systemic health in poultry (Schwartz and Vetvicka, 2021). Fermentation substrates, such as short-chain fatty acids (**SCFA**) being absorbed from the gut, are also known to act as important signaling molecules used for communication between the microbiota and host tissues (Ringseis et al., 2020). This communication is mediated by multiple SCFA receptors, which have been discovered in many metabolic tissues, including liver and skeletal muscle, and demonstrated to sense these microbiota-derived signals, thereby, affecting cellular signaling, gene expression and metabolic pathways. Additionally, due to their specific physicochemical properties, structural carbohydrates like β-glucans and chitin affect the digesta passage rate and may inhibit nutrient digestibility by increasing digesta viscosity and preventing nutrients from being enzymatically hydrolyzed (Wang et al., 1992).

Considering this, the present study aimed to comprehensively investigate the effects of feeding the fruiting bodies of *P. sapidus* on broiler performance, ileal nutrient digestibility, cecal microbiota composition, cecal integrity, liver transcriptome, and the expression of genes involved in protein turnover in breast muscle.

## Material and methods

### Animals, experimental design, and diets

The feeding trial with broilers was approved by the Animal Welfare Officer of the Justus Liebig University Giessen (approval no.: JLU 862_M). All experimental procedures adhered to established guidelines for the care and handling of laboratory animals. Seventy-two male, 1-day-old Cobb 500 broilers (Cobb-Vantress, Weidemar, Germany) were randomly assigned to three groups (4 broilers/cage, 6 cages/group) with similar mean initial body weights (BW) across all groups. Housing conditions, light regime, and temperature programs mirrored those of our recent study involving fungal mycelium (Schäfer et al., 2024a). The three groups were fed different nutrient-adequate diets containing either 0 g (group PSA-F0), 25 g (group PSA-F25), or 50 g (group PSA-F50) of freeze-dried *P. sapidus* fruiting bodies per kg diet, administered in a three-phase feeding system: a starter diet (days 1–10), a grower diet (days 11–21), and a finisher diet (days 22–35). The composition of these isoenergetic and isonitrogenic diets is presented in Table 1. The nutrient and energy composition of the diets met the broilers’ requirements, as recommended by the breeder (Cobb-Vantress, 2022). *P. sapidus* (DSMZ 8266) was propagated by Mycelia NV (Deinze, Belgium) to produce grain-based spawn. Wheat straw substrate, prepared by VeMe Specials BV (Gemert, Netherlands), was inoculated with *P. sapidus* spawn prior to being placed into micro-perforated substrate bags. These substrate bags were transported to Druid Austernpilze (Ottrau, Germany), where *P. sapidus* fruiting bodies were cultivated under standard commercial oyster mushroom production conditions (Rühl and Kües, 2007). Prior to mixing the *P. sapidus* fruiting bodies with the other feed components using mixing machines (V100 and V250, Diosna, Germany), the fruiting bodies were freeze-dried using a Gamma 1-20 freeze dryer (Christ, Osterode, Germany). Freeze-dried fruiting bodies were ground to pass a 1-mm sieve using a Retsch ZM1 centrifugal mill at 10,000 rpm (Retsch, Haan, Germany). Experimental diets were pelleted using a pelleting device (V3/30 C, Simon-Heesen, Boxtel, Netherlands) at approximately 70°C and 5 mPa extrusion pressure. In the first 3 days, the diet was offered in crumbled form, and afterward, until the end of the experiment, in pelleted form (2 mm pellet diameter). BW (individually) and feed intake (per cage) were recorded on days 1, 10, 21, and 35. The number of broilers alive was controlled daily and mortality during the whole period was calculated as the percentage of dead broilers at end of the experiment in relation to all broilers included at the start of the trial. The feed:gain ratio was calculated based on feed intake and BW gains of the broilers alive per cage.

**Table 1:** Composition of the broiler diets supplemented with either 0 g (PSA-F0), 25 g (PSA-F25) or 50 g (PSA-F50) *P. sapidus* fruiting bodies per kg diet.

	Starter diets			Grower diets			Finisher diets		
	PSA-F0	PSA-F25	PSA-F50	PSA-F0	PSA-F25	PSA-F50	PSA-F0	PSA-F25	PSA-F50
*Component (g/kg)*									
Maize	284.9	309.2	354.8	272.5	296.5	327.5	276.3	306.5	365.8
Soybean meal (42 % CP)	360.5	364.5	371	298.5	303.5	307.5	265.5	270.5	278.1
Wheat	166.6	145.8	98.5	228.6	207.6	177.3	250.9	222.6	161.1
*P. sapidus* fruiting bodies	0	25	50	0	25	50	0	25	50
Soybean oil	55	55	55	65	65	65	70	70	70
Wheat bran	62.5	30	0	63	30	0	62.5	30.5	0
Mineral & vitamin mix*	20	20	20	20	20	20	20	20	20
Monocalcium phosphate	15.2	15.5	16.5	16	16.5	17	15.8	16.6	17.5
Calcium carbonate	16	16	15.7	16	16	15.9	16.1	15.8	15.5
Sodium chloride	4	4	4	4	4	4	4	4	4
DL-Methionine	3.6	3.6	3.7	3.6	3.7	3.7	3.2	3.2	3.2
L-Lysine	3.7	3.6	3.4	4.3	4.1	4.0	3.6	3.4	3.2
L-Threonine	1.9	1.8	1.6	1.7	1.6	1.5	1.3	1.2	1.0
L-Arginine	2.4	2.2	2.0	2.4	2.2	2.2	2.0	2.0	2.0
L-Valine	2.5	2.5	2.4	2.8	2.7	2.7	2.2	2.2	2.2
L-Isoleucine	1.2	1.2	12	1.6	1.5	1.5	1.6	1.5	1.3
L-Tryptophan	0	0.1	0.2	0	0.1	0.2	0	0	0.1
Titanium dioxide	0	0	0	0	0	0	5	5	5

⁎ The mineral & vitamin mix supplied the following minerals and vitamins per kg diet (starter/grower/finisher): Fe, 40/40/40 mg; Cu, 15/15/15 mg; Mn, 100/100/100 mg; Zn, 100/100/100 mg; I, 1/1/1 mg; Se, 0.35/0.35/0.35 mg; vitamin A, 10,000/10,000/10,000 IU; vitamin D3, 5,000/5,000/5,000 IU; vitamin K3, 3/3/3 mg; vitamin E, 80/50/50 IU; vitamin B1, 3/2/2 mg; vitamin B2, 9/8/6 mg; vitamin B6, 4/3/3 mg; vitamin B12, 0.02/0.015/0.015 mg; biotin, 0.2/0.18/0.18 mg; folic acid, 2/2/1.5 mg; nicotinic acid, 60/50/50 mg; choline chloride, 500/400/350 mg; pantothenic acid, 15/12/10 mg.

### Sample collection

On day 35, all animals were euthanized by bleeding (*via* the *Vena jugularis* and *Arteria carotis*) under electrical anesthesia using a BTG-40A stunning device (Westerhoff Geflügeltechnik, Hoogstede, Germany), following European legislation for animal euthanasia (EUR_Lex, 09.01.2024). Twelve broilers per group (2 from each cage), representing the mean BW of the entire group, were selected for the determination of metabolic parameters. Plasma was prepared from whole blood, and aliquots from liver and breast muscle were collected, as previously described (Schäfer et al., 2024a, 2024b). In addition, the gastrointestinal tract was removed and digesta from the whole ileum (segment between Meckel´s diverticulum and the ileo-cecal junction) and the cecum collected *via* squeezing the respective intestinal segment. The cecum mucosa was obtained by scraping using a cell scraper following a washing step with ice-cold 0.9 % NaCl solution. All samples were immediately snap-frozen and stored at −80°C pending analysis.

### Chemical composition of fruiting bodies, other diet components and the diets

The concentrations of dry matter (**DM**), crude nutrients [crude protein **(CP**), ether extract (**EE**), crude ash (**CA**), crude fiber (**CF**)], sugars, starch, fatty acids, and amino acids in the fruiting bodies, main diet components (wheat, maize, soybean meal), and diets were analyzed according to official methods (Verband Deutscher Landwirtschaftlicher Untersuchungs- und Forschungsanstalten, VDLUFA, 2012). The CP content of the fruiting bodies was calculated using a *N*-to-protein conversion factor of 4.17, accounting for the presence of non-protein-*N* compounds (e.g., chitin) in the fruiting bodies (Souci et al., 2016). For other diet components, the CP content was calculated with a conversion factor of 6.25. Total, α-, and β-glucans in the fruiting bodies were determined using an enzymatic assay kit (Megazyme, Auchincruive, Scotland, UK). Chitin content was analyzed as described previously (Ahlborn et al., 2019; Maheshwari et al., 2020). The apparent *N*-corrected metabolizable energy (**AME*_N_***) content of the diets was calculated from crude nutrient contents (in g/kg diet) using the formula from the World’s Poultry Science Association for poultry compound feed (WPSA, 1984):

$$\text{AM}E_{N}\left(\text{MJ}/\text{kg}\right)=\left[\left(0.01551\cdot \text{CP}\right)+\left(0.03431\cdot \text{CL}\right)+\left(0.01669\cdot \text{Starch}\right)+\left(0.01301\cdot \text{Sugar}\right)\right]$$

### Apparent ileal digestibility of nutrients

The apparent ileal digestibility (**AID**) of EE, and amino acids at the end of the experiment was determined using the indicator method with titanium dioxide as an inert marker in the finisher diets (day 22–35), as previously described (Schäfer et al., 2024a).

### Microbiota diversity and composition in the cecal digesta

Extraction of total genomic DNA, PCR-based amplicon production, and 16S rRNA gene amplicon sequencing were carried out by Life & Brain GmbH (Bonn, Germany), following established protocols (Beller et al., 2024). The 16S sequencing data were processed using QIIME 2 version 2022.8 (Bolyen et al., 2019). Raw sequencing data were deposited as FASTQ files in the NCBI Sequence Read Archive repository under BioProject accession number PRJNA1236949. Sequencing quality control and denoising were performed using DADA2, amplicon sequencing variants (**ASV**) were identified using a pretrained SILVA classifier (silva-138-nr99-16S-V3-V4-classifier), and diversity metrics and microbial taxa abundance were analyzed using the MicrobiomeAnalyst platform (Beller et al., 2024).

### Concentrations of microbial fermentation products in the cecal digesta

Concentrations of SCFA in cecal digesta were measured using a Clarus 580 GC system (Perkin Elmer, Waltham, USA) equipped with a polar capillary column (30 m free fatty acid phase, 0.32 mm internal diameter, 0.25 μm film thickness; Macherey & Nagel, Düren, Germany) and a flame ionization detector, as previously described (Fiesel et al., 2014).

### Concentrations of endotoxins in plasma

Lipopolysaccharide (**LPS**) concentration in plasma was determined using the Chicken Lipopolysaccharide ELISA Kit from Assay Genie (Dublin, Ireland), following the manufacturer’s protocol.

### Total RNA extraction from liver, cecum mucosa and breast muscle and qPCR analysis

Total RNA was extracted from the liver (20–30 mg), cecum mucosa (30–50 mg), and breast muscle (30 mg) using TRIzol reagent (Invitrogen, Karlsruhe, Germany). Evaluation of RNA quantity and quality, synthesis of complementary DNA (cDNA), and quantitative PCR (qPCR) analysis were performed according to established procedures (Chiappisi et al., 2017; Meyer et al., 2019). Gene-specific primer pairs were designed using Primer2 and obtained from Eurofins MWG Operon (Ebersberg, Germany). Details of the primer pairs are provided in **Supplementary Table S1**. qPCR data were normalized using the following reference genes: *ACTB, GAPDH, SDHA*, and *YWHAZ* for liver; *ACTB, GAPDH*, and *YWHAZ* for cecum mucosa; and *ACTB, GAPDH, SDHA*, and *YWHAZ* for breast muscle (Vandesompele et al., 2002). Relative mRNA levels were calculated based on the comparative Ct method using the sample with the lowest Ct value as the reference, as described previously (Chiappisi et al., 2017).

### Concentrations of triglycerides and cholesterol in plasma and liver

Concentrations of triglycerides and cholesterol in plasma and liver lipid extracts were measured using enzymatic reagent kits (Triglycerides FS, Cholesterol FS; DiaSys Diagnostic Systems GmbH, Holzheim, Germany), following the protocols described previously (Schäfer et al., 2024a).

### Liver transcriptomics

Liver transcriptomics was conducted using Affymetrix GeneChip Array Chicken Gene 1.0 ST, which encompasses 18,214 genes. For transcriptome analysis, six liver total RNA samples per group were randomly selected and processed at the Genomics Core Facility “KFB - Center of Excellence for Fluorescent Bioanalytics” (Regensburg, Germany), following the Applied Biosystems GeneChip Whole Transcript (WT) PLUS Reagent Kit User Guide (Thermo Fisher Scientific, Waltham, MA, USA). Prior to processing, RNA quality was assessed using an Agilent 2100 Bioanalyzer (Agilent Technologies, Waldbronn, Germany). The RNA integrity number (RIN) averaged 7.44 ± 0.25 (*n* = 18, mean ± SD), indicating satisfactory RNA quality. Processing of the raw data (cell intensity files), calculation of summarized probe set signals (in log2 scale), comparison of fold changes (**FC**), and determination of significance (*P*-values) were performed as described previously (Beller et al., 2024). Gene names were annotated using the “ChiGene-1_0-st-v1.na36.galgal3.transcript.csv” annotation file. The microarray data have been made publicly available in MIAME-compliant format in the NCBI’s Gene Expression Omnibus (**GEO**) repository under accession number GSE288500 (Edgar et al., 2002). Differentially expressed transcripts were filtered based on FC > 1.3 or < −1.3 and *P*-value < 0.05 for comparisons between groups PSA-F50 vs. PSA-F0 and PSA-F25 vs. PSA-F0. Gene set enrichment analysis (**GSEA**) was performed on the identified differentially expressed transcripts to determine enriched Gene Ontology (**GO**) terms within the categories of biological process (**BP**), molecular function (**MF**), and cellular component (**CC**), using the Database for Annotation, Visualization, and Integrated Discovery bioinformatics resource version 6.8 (Huang et al., 2009; Sherman et al., 2022). GO terms were considered enriched if *P* < 0.05.

### Statistical analysis

Statistical analysis was conducted using SPSS 28 software (IBM, Armonk, NY, USA). The cage served as the experimental unit for performance metrics, while the individual animal was the unit for all other data. All parameters were tested for normal distribution using the Shapiro-Wilk test for performance metrics (cage-based, total *n* = 18 cages or *n* = 6 cages/group) and biochemical and qPCR data (two animals per cage, total *n* = 36 or *n* = 12 broilers/group). Homoscedasticity was assessed using Levene’s test. If normal distribution was achieved only after log-transformation, the log-transformed data were used for statistical analysis. Differences among the three groups were analyzed using one-way analysis of variance (**ANOVA**), followed by Tukey’s post-hoc test for normally distributed data with homogeneous variances. For data with heterogeneous variances, the means of the three groups were analyzed using Welch’s ANOVA with the Games-Howell post-hoc test. In cases where the data were not normally distributed, Kruskal-Wallis one-way ANOVA was applied, followed by the Mann-Whitney U test with Bonferroni correction as a post-hoc test. For all tests mentioned above, a *P*-value < 0.05 was considered statistically significant.

## Results

### Chemical composition of P. sapidus fruiting bodies and experimental diets

The *P. sapidus* fruiting bodies contained a gross energy content of 18.6 MJ and a DM content of 922 g per kg of fresh matter. The DM primarily consisted of *N*-free extract (559 g/kg DM), followed by CP (224 g/kg DM), CF (133 g/kg DM), CA (55.6 g/kg DM), and EE (29.3 g/kg DM). The concentrations of total, β-, and α-glucans and sugars were 506, 476, 30, and 73 g/kg DM, respectively. The chitin concentration was 61.2 g/kg DM. Concentrations of amino acids and the fatty acid composition of total lipids in *P. sapidus* fruiting bodies are provided in **Supplementary Table S2**. Glutamine/glutamic acid, alanine, and asparagine/aspartic acid had the highest concentrations of amino acids, while the levels of other amino acids were either below or slightly above 10 g/kg fresh matter. Tryptophan was the amino acid with the lowest concentration. The main fatty acids in total lipids were C18:2 n-6, followed by C18:1 n-9, C16:0, and C18:0. As shown in Table 2, the concentrations of crude nutrients (CP, EE, CA, CF), amino acids, AME*_N_*, and total lipid fatty acid composition were comparable across all three diets within each feeding phase.

**Table 2:** Nutrient and energy content of the broiler diets supplemented with either 0 g (PSA-F0), 25 g (PSA-F25) or 50 g (PSA-F50) *P. sapidus* fruiting bodies per kg diet.

	Starter diets			Grower diets			Finisher diets		
	PSA-F0	PSA-F25	PSA-F50	PSA-F0	PSA-F25	PSA-F50	PSA-F0	PSA-F25	PSA-F50
*Analyzed crude nutrient and energy content*									
DM (% FM)	87.6	87.4	87.4	87.6	88.4	88.1	87.8	88.3	88.3
CP (% DM)	24.2	23.8	24.3	22.8	22.7	22.6	21.2	20.9	20.9
EE (% DM)	8.9	9.2	9.4	10.0	9.6	9.9	10.5	10.6	10.5
CA (% DM)	6.9	6.8	6.8	6.5	6.5	6.3	6.8	6.7	6.7
CF (% DM)	5.2	4.9	4.8	4.8	4.3	4.4	4.7	4.3	4.3
Sugar (% DM)	3.6	4.1	4.4	2.5	3.7	3.8	2.2	3.0	3.0
Starch (% DM)	37.6	36.4	34.1	40.0	40.2	40.6	41.9	40.6	42.6
*Calculated energy content*									
AME*_N_* (MJ/kg DM)	13.5	13.5	13.2	14.0	14.0	14.2	14.2	14.0	14.3
Fatty acids (% of total fatty acids)*									
C16:0	12.3	12.5	12.5	12.2	12.3	12.4	11.8	12.0	12.0
C18:0	3.51	3.37	3.35	3.84	3.36	3.39	3.36	3.43	3.30
C18:1	20.8	20.5	20.4	21.4	20.1	20.3	21.3	21.1	20.8
C18:2	55.2	55.7	55.7	54.8	56.3	56.0	55.4	55.2	55.7
C18:3	5.09	5.00	5.11	5.30	5.27	5.08	5.33	5.12	5.30
C20:0	1.41	1.41	1.39	1.01	1.19	1.50	1.33	1.50	1.40
C20:1	0.29	0.30	0.38	0.31	0.31	0.29	0.21	0.31	0.32
C20:5	0.43	0.45	0.45	0.38	0.41	0.40	0.37	0.39	0.40
C22:0	0.26	0.26	0.27	0.27	0.23	0.25	0.38	0.36	0.37
C24:0	0.20	0.19	0.17	0.16	0.18	0.19	0.15	0.17	0.21
*Amino acids (g/kg diet)*									
Alanine	9.05	9.23	9.96	7.97	8.69	8.94	7.45	8.16	8.77
Arginine	14.70	14.06	14.48	13.22	13.34	12.96	11.80	12.08	12.17
Asparagine/Aspartic acid	18.37	17.83	18.57	15.75	16.50	16.08	14.22	14.99	15.23
Cysteine	3.49	3.31	3.29	3.01	3.16	3.37	2.96	2.90	3.26
Glutamine/Glutamic acid	35.02	33.64	34.34	31.81	32.52	31.10	30.98	30.40	29.78
Glycine	8.06	7.80	8.04	7.21	7.32	7.08	6.69	6.95	6.86
Histidine	4.83	4.68	4.80	4.21	4.46	4.15	3.90	3.99	4.10
Isoleucine	8.43	8.10	8.58	8.08	8.00	7.90	7.40	7.56	7.32
Leucine	15.23	15.26	16.11	14.05	14.50	14.29	13.04	13.64	13.84
Lysine	13.06	12.66	13.08	11.86	12.10	11.82	10.48	10.81	10.80
Methionine	6.60	6.38	6.46	5.72	6.32	6.36	5.53	5.40	6.00
Phenylalanine	9.25	8.95	9.46	8.28	8.58	8.33	7.64	7.93	8.01
Proline	11.91	11.09	11.44	10.98	10.89	10.52	10.15	10.01	10.54
Serine	10.35	10.02	10.28	8.92	9.35	9.00	8.22	8.56	8.80
Threonine	9.52	9.07	9.17	8.08	8.49	8.24	7.33	7.51	7.72
Tryptophan	2.50	2.66	2.58	2.12	2.39	2.10	1.65	2.05	2.15
Tyrosine	5.51	5.16	5.61	4.90	4.80	4.62	4.29	4.52	4.45
Valine	9.90	9.56	9.92	9.43	9.35	9.37	8.21	8.56	8.57

⁎ Only fatty acids > 0.1 % of total fatty acids are shown. Abbreviations: CA, crude ash; CF, crude fiber; CP, crude protein; DM, dry matter; EE, ether extract; FM, fresh matter.

### Growth performance, carcass characteristics and AID of nutrients

Final BW, BW gain over the entire period, and BW gain during the finisher period were significantly lower in groups PSA-F25 and PSA-F50 compared to group PSA-F0 (*P* < 0.05, Table 3). BW gain during the starter period (*P* = 0.055) and grower period (*P* = 0.066) did not differ among the groups. Feed intake over the entire period and during each feeding phase was not significantly different between groups. The feed:gain gain ratio during the entire period (*P* = 0.858), grower period (*P* = 0.230), and finisher period (*P* = 0.780) showed no significant differences among groups but was higher in group PSA-F25 than in group PSA-F0 during the starter period (*P* < 0.05). Mortality rates over the entire period were 8.4 % in group PSA-F0 and 4.2 % in groups PSA-F25 and PSA-F50. Weights of the eviscerated carcass, breast muscle, and thigh muscle were lower in group PSA-F50 compared to group PSA-F0 (*P* < 0.05, Table 4), whereas no significant differences were observed between groups PSA-F25 and PSA-F0 or PSA-F50 and PSA-F25. Dressing percentage (*P* = 0.088), relative breast muscle weight (*P* = 0.058), and relative thigh muscle weight (*P* = 0.070) did not differ significantly among groups. The AID values for EE (*P* = 0.483) and amino acids (lowest *P*-value: 0.057 for glutamine/glutamic acid, highest *P*-value: 0.995 for methionine) were not significantly different among groups. However, the AID values for glutamine/glutamic acid (*P* = 0.057) and cysteine (*P* = 0.097) showed a tendency to be reduced in group PSA-F50 compared to the other groups (Table 5). The average daily apparently digested amounts of amino acids during the finisher period were calculated from daily feed intake during the finisher period, dietary concentrations of amino acids in the finisher period and the AID of amino acids determined at the last day of the finisher period. Due to lack of data for the other days of the finisher period, the same AID of amino acids was assumed for all days of the finisher period. Amongst the indispensable amino acids, the apparently digested amounts of arginine, isoleucine, leucine, lysine, phenylalanine, threonine and valine were lower in group PSA-F50 than in group PSA-F0 (*P* < 0.05), whereas no difference was seen between groups PSA-F25 and PSA-F0 with regard to these amino acids (Table 6). The apparently digested amount of methionine was lower in group PSA-F25 than in group PSA-F0 (*P* < 0.05), but did not differ between groups PSA-F50 and PSA-F0. The apparently digested amount of tryptophan was higher in group PSA-F50 than in group PSA-F0 (*P* < 0.05), but was not different between group PSA-F25 and group PSA-F0. The apparently digested amount of histidine did not differ across groups. Amongst the dispensable amino acids, the apparently digested amounts glutamine/glutamic acid, glycine, proline, serine and tyrosine were lower in group PSA-F50 than in group PSA-F0 (*P* < 0.05), whereas no difference – with the exception of proline – was observed between groups PSA-F25 and PSA-F0. The apparently digested amount of cysteine was lower in group PSA-F25 than in group PSA-F0 but was not different between group PSA-F50 and PSA-F0. The apparently digested amounts of alanine and asparagine/aspartic acid were not different among groups.

**Table 3:** Performance data of broilers fed diets with either 0 g (PSA-F0), 25 g (PSA-F25) or 50 g (PSA-F50) *P. sapidus* fruiting bodies per kg diet for 35 days.

	PSA-F0	PSA-F25	PSA-F50	*P*-value
*Whole period (day 1 to 35)*				
Initial BW (g)	45.7 ± 1.9	45.7 ± 1.7	45.7 ± 1.8	0.938
Final BW (g)	2992 ± 384^a^	2720 ± 497^b^	2672 ± 435^b^	0.016
BW gain (g)	2946 ± 385^a^	2675 ± 497^b^	2626 ± 435^b^	0.016
Feed intake (g)	4104 ± 338	3674 ± 356	3732 ± 200	0.079
Feed:gain ratio (g/g)	1.39 ± 0.16	1.37 ± 0.03	1.43 ± 0.11	0.858
Mortality (%)	8.4	4.2	4.2	-
*Starter period (day 1 to 10)*				
BW gain (g)	284 ± 18	269 ± 23	274 ± 25	0.055
Feed intake (g)	290 ± 4	285 ± 12	288 ± 9	0.618
Feed:gain ratio (g/g)	1.02 ± 0.02^b^	1.06 ± 0.01^a^	1.05 ± 0.02^ab^	0.011
*Grower period (day 11 to 21)*				
BW gain (g)	904 ± 59	850 ± 108	867 ± 79	0.066
Feed intake (g)	1106 ± 17	1066 ± 79	1086 ± 37	0.456
Feed:gain ratio (g/g)	1.22 ± 0.04	1.25 ± 0.02	1.25 ± 0.03	0.230
*Finisher period (day 22 to 35)*				
BW gain (g)	1760 ± 360^a^	1556 ± 428^b^	1483 ± 375^b^	0.016
Feed intake (g)	2708 ± 331	2323 ± 281	2359 ± 188	0.072
Feed:gain ratio (g/g)	1.55 ± 0.27	1.49 ± 0.07	1.60 ± 0.19	0.780

Data with the exception of mortality are means ± SD, *n* = 6 cages/group. ^a,b^Means without a common letter differ across the groups, *P* < 0.05. Abbreviation: BW, body weight.

**Table 4:** Carcass characteristics of broilers fed diets with either 0 g (PSA-F0), 25 g (PSA-F25) or 50 g (PSA-F50) *P. sapidus* fruiting bodies per kg diet for 35 days.

	PSA-F0	PSA-F25	PSA-F50	*P*-value
Eviscerated carcass weight (g)	2139 ± 261^a^	1964 ± 411^ab^	1856 ± 393^b^	0.026
Dressing percentage (%)	72.5 ± 3.5	71.8 ± 3.2	69.0 ± 6.3	0.088
Breast muscle weight				
Absolute (g)	649 ± 98^a^	568 ± 138^ab^	539 ± 138^b^	0.014
Relative (% of BW)	21.7 ± 2.3	20.4 ± 2.6	19.9 ± 2.8	0.058
Thigh muscle weight				
Absolute (g)	582 ± 77^a^	545 ± 119^ab^	505 ± 104^b^	0.039
Relative (% of BW)	19.5 ± 1.8	20.0 ± 1.0	18.8 ± 1.8	0.070

Data are means ± SD, *n* = 22-23 broilers/group. ^a,b^Means without a common letter differ across the groups, *P* < 0.05.

**Table 5:** Apparent ileal digestibility (AID) of ether extract (EE) and amino acids of broilers fed diets with either 0 g (PSA-F0), 25 g (PSA-F25) or 50 g (PSA-F50) *P. sapidus* fruiting bodies per kg diet for 35 days.

	PSA-F0	PSA-F25	PSA-F50	*P*-value
AID of EE (%)	87.1 ± 4.3	87.6 ± 2.5	85.4 ± 6.1	0.483
AID of amino acids (%)				
Indispensable amino acids				
Arginine	86.7 ± 3.3	87.7 ± 2.2	86.5 ± 2.4	0.498
Histidine	81.7 ± 4.3	82.6 ± 3.3	80.9 ± 3.6	0.559
Isoleucine	83.5 ± 4.3	84.1 ± 3.5	80.8 ± 3.2	0.107
Leucine	81.9 ± 4.2	83.2 ± 3.4	80.4 ± 3.7	0.246
Lysine	85.2 ± 4.4	86.0 ± 3.6	84.2 ± 2.8	0.528
Methionine	91.7 ± 2.8	92.0 ± 2.0	91.9 ± 2.4	0.955
Phenylalanine	82.1 ± 4.4	83.5 ± 3.3	80.9 ± 3.5	0.292
Threonine	78.1 ± 6.0	79.1 ± 4.3	75.9 ± 5.1	0.260
Tryptophan	81.7 ± 6.5	83.9 ± 4.3	84.1 ± 6.9	0.590
Valine	83.8 ± 4.3	85.0 ± 3.1	82.3 ± 3.4	0.241
Dispensable amino acids				
Alanine	73.2 ± 8.0	73.9 ± 5.9	68.3 ± 7.8	0.104
Asparagine/Aspartic acid	80.5 ± 4.0	81.4 ± 3.3	79.3 ± 3.0	0.366
Cysteine	74.7 ± 5.0	74.0 ± 5.6	69.7 ± 6.7	0.097
Glutamine/Glutamic acid	86.6 ± 2.8	86.2 ± 3	83.7 ± 3.0	0.057
Glycine	76.3 ± 5.2	77.6 ± 4.0	74.1 ± 4.7	0.226
Proline	83.2 ± 3.7	83.5 ± 3.0	80.9 ± 4.4	0.198
Serine	78.5 ± 5.4	79.9 ± 4.1	76.5 ± 4.8	0.264
Tyrosine	80.7 ± 5.5	81.9 ± 3.7	78.7 ± 3.9	0.254

Data are means ± SD, *n* = 12 broilers/group.

**Table 6:** Average daily apparently digested amounts of amino acids in broilers fed diets with either 0 g (PSA-F0), 25 g (PSA-F25) or 50 g (PSA-F50) *P. sapidus* fruiting bodies per kg diet during the finisher period.

	PSA-F0	PSA-F25	PSA-F50	*P*-value
Indispensable amino acids (g/day)				
Arginine	1.96 ± 0.23^a^	1.77 ± 0.23^ab^	1.73 ± 0.15^b^	0.024
Histidine	0.61 ± 0.08	0.55 ± 0.08	0.55 ± 0.06	0.063
Isoleucine	1.19 ± 0.15^a^	1.06 ± 0.15^ab^	0.95 ± 0.07^b^	0.001
Leucine	2.05 ± 0.25^a^	1.90 ± 0.27^ab^	1.80 ± 0.14^b^	0.041
Lysine	1.71 ± 0.21^a^	1.56 ± 0.21^ab^	1.49 ± 0.14^b^	0.023
Methionine	0.97 ± 0.11^a^	0.83 ± 0.11^b^	0.91 ± 0.08^ab^	0.009
Phenylalanine	1.20 ± 0.15^a^	1.11 ± 0.16^ab^	1.06 ± 0.10^b^	0.047
Threonine	1.10 ± 0.14^a^	1.00 ± 0.14^ab^	0.96 ± 0.11^b^	0.036
Tryptophan	0.24 ± 0.03^b^	0.27 ± 0.04^ab^	0.29 ± 0.03^a^	0.005
Valine	1.32 ± 0.16^a^	1.22 ± 0.17^ab^	1.13 ± 0.08^b^	0.014
Dispensable amino acids (g/day)				
Alanine	1.04 ± 0.15	1.01 ± 0.16	1.00 ± 0.14	0.719
Asparagine/Aspartic acid	2.20 ± 0.27	1.98 ± 0.33	1.99 ± 0.18	0.106
Cysteine	0.41 ± 0.04^a^	0.36 ± 0.05^b^	0.37 ± 0.04^ab^	0.013
Glutamine/Glutamic acid	4.93 ± 0.63^a^	4.39 ± 0.60^ab^	4.10 ± 0.38^b^	0.003
Glycine	0.98 ± 0.12^a^	0.90 ± 0.13^ab^	0.81 ± 0.07^b^	0.004
Proline	1.62 ± 0.19^a^	1.40 ± 0.19^b^	1.37 ± 0.11^b^	0.002
Serine	1.24 ± 0.16^a^	1.15 ± 0.17^ab^	1.08 ± 0.09^b^	0.035
Tyrosine	0.66 ± 0.08^a^	0.62 ± 0.09^ab^	0.57 ± 0.07^b^	0.020

Data are means ± SD, *n* = 12 broilers/group. ^a,b^Means without a common letter differ across the groups, *P* < 0.05.

### Diversity and composition of the cecum microbiota

**Fig. 1:**
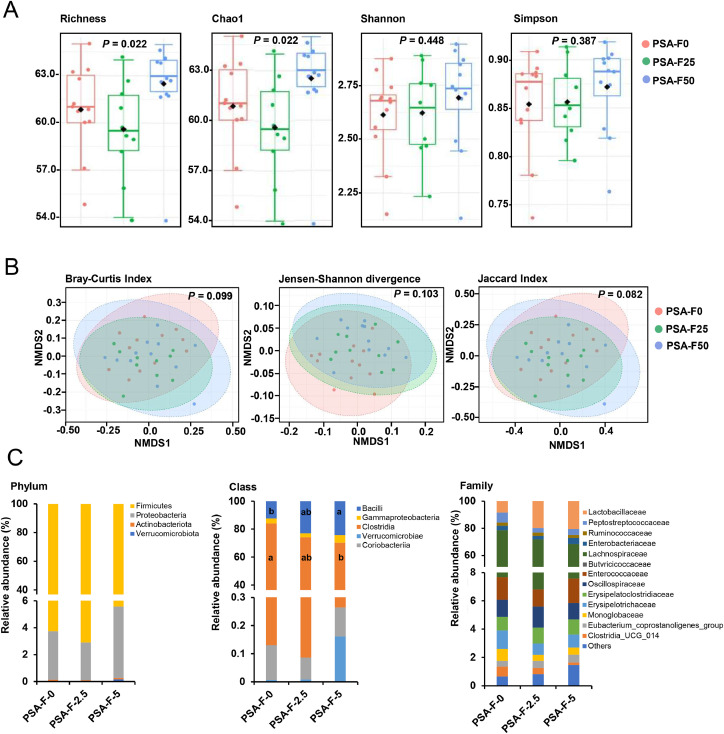
Analysis of the cecal microbiota. Indicators of α-diversity (Richness, Chao1, Shannon Index and Simpson Index) of the cecal bacterial community (A), visualization of the difference in the β-diversity (Bray-Curtis Index, Jenson-Shannon divergence and Jaccard Index) of cecal community between the groups by NMDS plots (B) and taxonomic composition (phylum, order, family) (C) of the cecal bacteria of broilers fed diets with either 0 g (PSA-F0), 25 g (PSA-F25) or 50 g (PSA-F50) *P. sapidus* fruiting bodies for 35 days. A: box plot for *n* = 12 broilers/group; B: NMDS plots for *n* = 12 broilers/group; C: Data are means for *n* = 12 broilers/group.

Following data normalization and filtering, 71 ASV were used to analyze cecal microbiota diversity and composition. The effect on α-diversity was assessed by calculating Chao1, Richness, Shannon Index, and Simpson Index (Fig. 1**A**). The species richness indicators, Chao1 and Richness, were significantly different among groups (both *P* = 0.022); both were higher in group PSA-F50 compared to group PSA-F25 (*P* = 0.016) and group PSA-F0 (*P* = 0.038), but no differences were observed between groups PSA-F25 and PSA-F0. The Shannon Index and Simpson Index did not differ among groups. The β-diversity metrics (Bray-Curtis Index, Jensen-Shannon Divergence, Jaccard Index) also showed no differences among groups, as visualized by non-metric multidimensional scaling (NMDS) plots (Fig. 1**B**). The impact of dietary treatment on cecal microbiota composition was systematically analyzed across taxonomic levels (phylum, class, order, family, genus, species) (Fig. 1**C**). Cecal bacteria in all groups belonged to four phyla, predominantly Firmicutes (94.4–97.0 % in all groups), followed by Proteobacteria (2.9–5.3 %), Actinobacteriota (0.09–0.12 %), and Verrucomicrobiota (0.01–0.16 %). The abundance of bacterial phyla did not differ among groups. At the class level, five taxa were identified (Bacilli, Clostridia, Coriobacteriia, Gammaproteobacteria, Verrucomicrobiae), with differences seen across groups in the abundance of Clostridia and Bacilli. Clostridia abundance was lower in group PSA-F50 than in group PSA-F0 (*P* = 0.003), while Bacilli abundance was higher in group PSA-F50 than in group PSA-F0 (*P* = 0.005). No differences were observed between groups PSA-F25 and PSA-F0 or PSA-F50 and PSA-F25. Cecal bacteria were classified into 14 orders, with Lachnospirales (58.9–68.7 %), Lactobacillales (10.1–22.3 %), Oscillospirales (5.8–7.3 %), and Peptostreptococcales_Tissierellales (3.5–7.3 %) being dominant. No differences in the abundance of these orders were found among groups. At the family level, 22 taxa were identified, primarily Lachnospiraceae (58.9–68.7 %), Lactobacillaceae (8.5–20.6 %), and Peptostreptococcaceae (3.5–7.3 %). Again, no significant differences were observed among groups. At the genus level, 54 bacterial taxa were identified, and their abundance did not differ across groups. At the species level, 71 taxa were identified, with only one species (*unknown_Erysipelatoclostridium*) showing differences in abundance among groups. Its abundance was higher in group PSA-F50 compared to group PSA-F25 (*P* = 0.008), and higher in group PSA-F25 compared to group PSA-F0 (*P* = 0.007). The abundance of all bacterial taxa in cecal digesta is detailed in **Supplemental Table S3.**

### Cecal digesta concentrations of SCFA

**Fig. 2:**
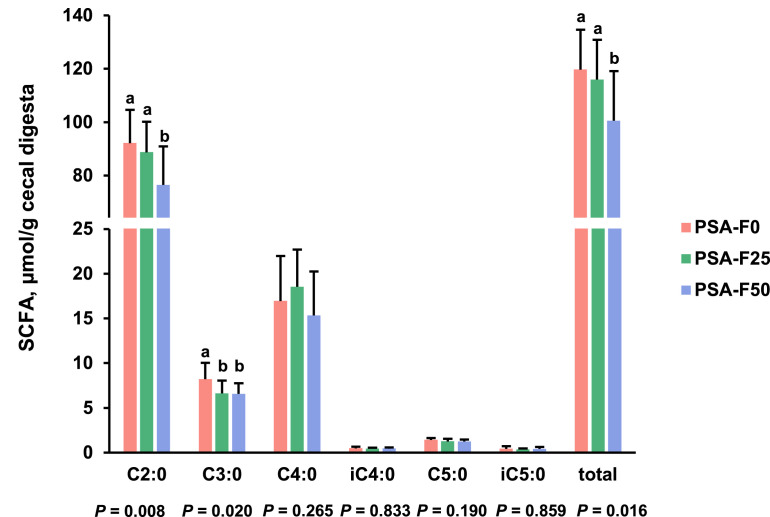
Concentrations of individual (acetic acid (C2:0); propionic acid (C3:0); butyric acid (C4:0); isobutyric acid (iC4:0); valeric acid (C5:0); isovaleric acid (iC5:0)) and total (= sum of all individual) short-chain fatty acids (SCFA) in cecal digesta of broilers fed diets with either 0 g (PSA-F0), 25 g (PSA-F25) or 50 g (PSA-F50) *P. sapidus* fruiting bodies for 35 days. Data are means ± SD, *n* = 12 broilers/group.

The concentrations of acetic acid and total SCFA in cecal digesta were lower in group PSA-F50 compared to groups PSA-F25 and PSA-F0 (*P* < 0.05, Fig. 2). Similarly, the concentration of propionic acid was lower in group PSA-F50 than in group PSA-F0, and in group PSA-F25 than in group PSA-F0 (*P* < 0.05). However, no differences were observed between groups PSA-F50 and PSA-F25. The concentrations of butyric acid, isobutyric acid, valeric acid, and isovaleric acid in cecal digesta were not different among groups. Relative concentrations of individual SCFA did not differ among groups (data not shown).

### Concentration of LPS in plasma and mRNA levels of genes involved in gut integrity in the cecum mucosa

Plasma LPS concentration did not differ among the three groups (Table 7). mRNA levels of *CLDN3, MUC2*, and *MUC5AC* were higher in group PSA-F50 compared to group PSA-F0 (*P* < 0.05). mRNA levels of *CLDN5, OCLN, MUC13*, and proinflammatory genes (*IL1B, IL8L1, IL8L2, TLR4, VCAM1*) in cecum mucosa were not significantly different among groups (Table 7).

**Table 7:** Concentrations of lipopolysaccharides in plasma and mRNA levels of genes in cecum mucosa of broilers fed diets with either 0 g (PSA-F0), 25 g (PSA-F25) or 50 g (PSA-F50) *P. sapidus* fruiting bodies per kg diet for 35 days.

	PSA-F0	PSA-F25	PSA-F50	*P*-value
Lipopolysaccharides (pg/ml)	155 ± 138	173 ± 153	109 ± 101	0.488
Relative mRNA levels (fold of PSA-F0)				
*Tight-junction proteins*				
*CLDN3*	1.00 ± 0.67^b^	2.19 ± 1.92^ab^	2.63 ± 1.73^a^	0.046
*CLDN5*	1.00 ± 0.86	0.68 ± 0.20	1.07 ± 0.56	0.355
*OCLN*	1.00 ± 0.51	1.01 ± 0.77	1.28 ± 0.71	0.559
*Mucins*				
*MUC2*	1.00 ± 0.89^b^	1.98 ± 0.75^a^	4.56 ± 3.28^a^	0.003
*MUC5AC*	1.00 ± 0.78^b^	1.31 ± 0.79^b^	3.28 ± 1.98^a^	0.006
*MUC13*	1.00 ± 0.73	1.18 ± 0.75	1.60 ± 1.14	0.284
*Proinflammatory genes*				
*IL1B*	1.00 ± 0.91	1.33 ± 0.85	1.63 ± 0.78	0.217
*IL8L1*	1.00 ± 0.81	1.85 ± 1.28	1.83 ± 1.29	0.107
*IL8L2*	1.00 ± 0.61	1.29 ± 0.63	1.18 ± 0.9	0.414
*TLR4*	1.00 ± 0.82	1.13 ± 0.83	1.43 ± 0.8	0.366
*TNFA*	1.00 ± 0.86	1.21 ± 0.77	1.93 ± 1.76	0.201
*VCAM1*	1.00 ± 0.84	0.98 ± 0.66	1.27 ± 0.71	0.492

Data are means ± SD, *n* = 12 broilers/group. ^a,b^Means without a common letter differ across the groups, *P* < 0.05.

### Liver weight and concentrations of triglycerides and cholesterol in liver and plasma

Absolute and relative liver weights, hepatic concentrations of triglycerides and cholesterol, and plasma triglyceride concentration did not differ among the groups (**Supplemental Table S4**). Plasma cholesterol concentration was lower in group PSA-F25 compared to group PSA-F0 (*P* < 0.05) but showed no differences between groups PSA-F50 and PSA-F0 or PSA-F50 and PSA-F25.

### Expression of genes involved in inflammation and protein turnover in breast muscle

The mRNA levels of various genes related to key pathways in protein synthesis and degradation—including the mammalian target of rapamycin (mTOR) pathway (*MTOR, S6K1, 4EBP1*), myogenesis (*MYF5, MYOD1, MYOG*), the ubiquitin-proteasome system (*FBXO32, FOXO1, MURF1*), the autophagy-lysosomal pathway (*ATG5, ATG9A, BECN1*), and the general control nonderepressible 2 (GCN2)/eukaryotic translation initiation factor 2A (*eIF2A*) pathway (*SQSTM1*)—did not vary across the three groups (**Supplemental Table S5**). Similarly, mRNA levels of inflammatory genes (*IL8L1, TNFA, TLR4, VCAM1*) were not different across groups (**Supplemental Table S5**).

### Differential gene expression in the liver

**Fig. 3:**
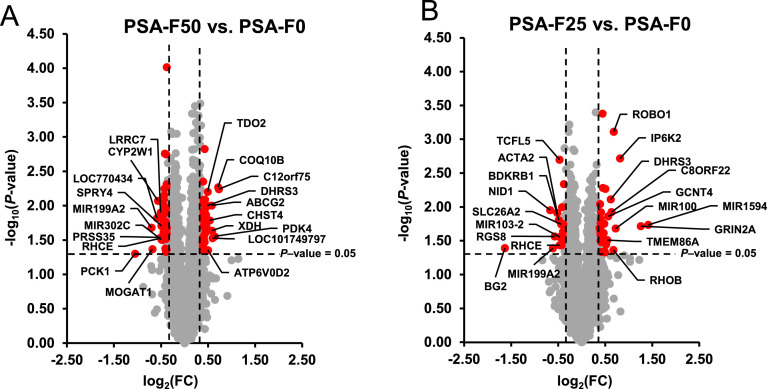
Differential transcriptome analysis in the liver (*n* = 6 microarrays/group). Volcano plot illustrating the differentially expressed transcripts in the liver of broilers between group PSA-F50 vs. PSA-F0 (A) and group PSA-F25 vs. PSA-F0 (B). The filtering criteria are indicated by horizontal (*P*-value = 0.05) and vertical (fold change >1.3 or < −1.3) dashed lines. Red dots in the upper left and upper right corner represent the downregulated and upregulated transcripts.

A total of 66 transcripts were identified as differentially expressed in the liver between groups PSA-F50 and PSA-F0 based on the filter criteria (FC > 1.3 and FC < −1.3, *P* < 0.05). Of these, 32 transcripts were upregulated and 34 were downregulated. Only five and four transcripts were regulated > 1.5-fold and < −1.5-fold, respectively. In Fig. 3**A**, the differentially expressed transcripts between groups PSA-F50 and PSA-F0 are displayed as red dots in the volcano plot, with the top 10 upregulated and downregulated transcripts labeled by their gene symbols. The FC and *P*-values of all differentially expressed transcripts are provided in **Supplemental Table S6**, along with the corresponding data for the same genes in the comparison PSA-F25 vs. PSA-F0. Among these transcripts, 13 (*ABCG2, DHRS3, FAM3B, YPEL5, TLR5, SH3BP4L, LOC415787, MIR551B, METTL21EP, SLC25A4, TCFL5, RHCE* and *MIR199A2*) were significantly regulated in both group comparisons (PSA-F50 vs. PSA-F0 and PSA-F25 vs. PSA-F0). A total of 47 transcripts were found to be differentially expressed between groups PSA-F25 and PSA-F0, with 30 upregulated and 17 downregulated. Eight transcripts were regulated > 1.5-fold, and three transcripts were regulated < −1.5-fold. Differentially expressed transcripts between groups PSA-F25 and PSA-F0 are shown in Fig. 3**B** as red dots in the volcano plot, with the top 10 upregulated and downregulated transcripts labeled. The FC and *P*-values of all transcripts are detailed in **Supplemental Table S7.**

For validation, the expression of 18 transcripts that were differentially expressed in the comparison PSA-F50 vs. PSA-F0 (according to microarray analysis) was measured by qPCR. **Supplemental Table S8** demonstrates that the effect direction (positive or negative FC) was consistent between microarray and qPCR analyses for all validated transcripts. However, the effect size (FC value) showed slight variations between the methods. Statistical analysis of qPCR-based FC revealed significant differences (*P* < 0.05) only for two transcripts (*ABCG2* and *CYP2W1*) in the comparison PSA-F50 vs. PSA-F0, while no significant differences were observed for the other 16 transcripts (*P* ≥ 0.05).

GSEA within the three GO categories (BP, MF, CC) identified 22 enriched GO terms (*P* < 0.05) among the 32 upregulated transcripts in the comparison PSA-F50 vs. PSA-F0. The most enriched terms included potassium channel regulator activity, voltage-gated potassium channel complex, potassium channel complex, transmembrane transporter complex, and extracellular space (lowest EASE scores). Using the 34 downregulated transcripts from the same comparison, GSEA revealed 29 enriched GO terms (*P* < 0.05), such as protein localization to the plasma membrane, protein localization to the cell periphery, glycerol metabolic process, neurotransmitter receptor transport (endosome to postsynaptic membrane), and neurotransmitter receptor transport (endosome to plasma membrane). All enriched GO terms for upregulated and downregulated transcripts are listed in **Supplemental Table S9**. GSEA was not performed for the comparison PSA-F25 vs. PSA-F0 due to the low number of differentially expressed transcripts in this comparison.

## Discussion

To evaluate the effects of dietary *P. sapidus* fruiting bodies on broiler performance and metabolism*, P. sapidus* fruiting bodies were incorporated into nutritionally adequate diets by replacing wheat and wheat bran at two inclusion levels (25 and 50 g/kg diet). These inclusion levels were selected to compare the effects of *P. sapidus* fruiting bodies with those of *P. sapidus* mycelium observed in our recent studies (Schäfer et al., 2024a, 2024b). To ensure comparable crude nutrient and AME*_N_* levels across the three diets in each phase, as confirmed through chemical analysis, the proportions of maize and soybean meal were increased with higher inclusion levels of *P. sapidus* fruiting bodies. Consequently, the effects observed in the two groups fed *P. sapidus* fruiting bodies cannot be solely attributed to the fruiting body constituents but may also result from changes in diet composition. One relevant factor in this regard is the partial or complete replacement of wheat bran in the diets of groups PSA-F25 and PSA-F50, respectively, which was necessary to offset the high fiber content of the fruiting bodies. However, it should be noted that the fiber fraction of wheat bran differs significantly from fungal biomass, as different fibers exhibit distinct physicochemical properties such as solubility, viscosity, binding capacity, bulking ability, and fermentability (Singh and Kim, 2021). Another factor arising from the altered diet composition is the higher soybean meal content in diet PSA-F50 compared to diets PSA-F25 and PSA-F0. Since soybean meal contains various bioactive compounds, including isoflavones, soyasaponins, and phenolics like flavonoids (Kao et al., 2008; De Oliveira Silva and Perrone, 2015), which possess antioxidant, anti-inflammatory, and other biological activities, it cannot be ruled out that the effects observed in groups PSA-F50 and PSA-F25 may be partially due to the increased intake of these bioactive compounds.

A key finding of the present study is that broilers fed diets containing 25 and 50 g/kg of *P. sapidus* fruiting bodies exhibited lower final BW and BW gains over the finisher and the entire period compared to broilers fed diets without fruiting bodies. Although feed intake during the finisher and the entire period did not statistically differ among groups, it tended to be lower during both the entire and finisher period in broilers fed *P. sapidus* fruiting bodies compared to those fed no fruiting bodies. Consequently, the numerically lower feed intake in the two groups fed *P. sapidus* fruiting bodies explains why the feed:gain ratio did not differ among groups. The reason for the numerically lower feed intake in broilers fed *P. sapidus* fruiting bodies during the finisher phase remains unclear. However, it may be attributed to differences in diet composition and the resulting fiber supply between the groups. As discussed above, the physicochemical properties of the fiber fractions from different sources, such as wheat bran and fruiting bodies, differ markedly. Thus, it is possible that inclusion of the fruiting bodies in the diet increased digesta volume and viscosity, potentially enhancing satiety and thereby lowering feed intake. Despite that systemic availability of SCFA from fiber fermentation plays an important role in the regulation of food intake through inducing satiety (Xiong et al., 2004), an increased systemic availability of SCFA in broilers fed the fruiting bodies is unlikely to explain the reduced feed intake, because total SCFA concentration in the cecum digesta of these broilers was even reduced. Another possibility is that the dietary inclusion of the fruiting bodies impaired feed palatability, consequently reducing intake.

Additionally, eviscerated carcass weight and the weights of the most important meat cuts - breast and thigh - were lower in the group fed the higher dose of *P. sapidus* fruiting bodies compared to the group fed no fruiting bodies. In the group fed the lower dose of *P. sapidus* fruiting bodies, eviscerated carcass, breast muscle, and thigh muscle weights were also notably lower than those in the group fed no fruiting bodies; however, these differences were not statistically significant. Therefore, the performance and slaughter data from the present study clearly indicate that dietary inclusion of *P. sapidus* fruiting bodies at 25 and 50 g/kg diet impairs broiler performance. This contrasts with findings from our recent study on *P. sapidus* mycelium, where the same inclusion levels (25 and 50 g/kg diet) did not negatively affect growth performance in Cobb 500 broilers (Schäfer et al., 2024a). One notable difference between *P. sapidus* fruiting bodies and mycelium is their β-glucan content, which was found to be higher in the fruiting bodies (47.6 % of DM) than in the vegetative mycelium (31.2 % of DM; Schäfer et al., 2024a). Considering the established inhibitory effects of high β-glucan levels on nutrient digestibility (Wang et al., 1992), the observed differences in broiler performance between the current study and our previous study (Schäfer et al., 2024a) may be attributed to variations in dietary β-glucan concentrations and their impact on nutrient digestibility. Consistent with this explanation, AID of CP, EE, and all individual amino acids was unaffected by dietary inclusion of *P. sapidus* mycelium in our previous study (Schäfer et al., 2024a). In contrast, in the present study, the AID of EE, and most individual amino acids was numerically lower - or tended to be lower (cysteine, glutamine/glutamic acid) - in the group fed the higher dose of *P. sapidus* fruiting bodies compared to the group fed no fruiting bodies. Taking into account the reduced feed intake in broilers fed the fruiting bodies during the finisher period, the apparently digested amounts of most amino acids including the indispensable amino acids arginine, isoleucine, leucine, lysine, phenylalanine, threonine and valine during the finisher period were even significantly reduced in these broilers. Thus, a decreased availability of indispensable amino acids could provide a plausible explanation for the decreased BW gain during the finisher period and the decreased eviscerated carcass weight and breast and thigh muscle weights in broilers fed the high dose of the fruiting bodies.

The higher chitin content in *P. sapidus* fruiting bodies compared to mycelium (5.3 % of DM; Schäfer et al., 2024a) may have also contributed to this effect, as chitin has been shown to negatively affect nutrient digestibility in broilers (Razdan and Pettersson, 1994). In addition, evidence has been gained that chitosan, which is formed from chitin by partial deacetylation, reduces feed intake in both mice (Kumar et al., 2009) and pigs (Walsh et al., 2013) through altering serum levels and/or gene expression levels of important appetite- and satiety-regulating signals, such as leptin, neuromedin B and neuropeptide Y (Egan et al., 2016). Collectively, these findings suggest that the combined reduction in feed intake and nutrient digestibility likely contributed to the decreased growth performance observed in broilers fed *P. sapidus* fruiting bodies.

A further finding of the present study is that the cecal microbiota structure was only marginally influenced by the dietary inclusion of *P. sapidus* fruiting bodies. This was evident from a systematic analysis of the taxonomic composition of the cecal microbiota, which showed that only the abundance of one minor species, namely *unknown Erysipelatoclostridium*, out of 71 bacterial species identified, differed among groups. Additionally, while two α-diversity metrics, Chao1 and Richness, were higher in broilers fed the higher dose of *P. sapidus* fruiting bodies compared to those fed no fruiting bodies, the other two α-diversity metrics (Shannon Index and Simpson Index), which also account for evenness, as well as all β-diversity metrics evaluated (Bray-Curtis Index, Jensen-Shannon Divergence, Jaccard Index), showed no differences among groups. Overall, these observations suggest that the inclusion of *P. sapidus* fruiting bodies in broiler diets has a very low impact on cecal microbiota composition and diversity. In this regard, the effects of the fruiting bodies are similar to those of *P. sapidus* mycelium observed in our recent study, where the cecal microbiota community structure was also only marginally affected (Schäfer et al., 2024a). Although the impact of fruiting bodies on the cecal microbiota was limited, future studies should also consider their potential effects on the microbiota of the small intestine. Given the known influence of β-glucans on digesta viscosity and passage rate through the small intestine, it is conceivable that the composition of the small intestinal microbiota was affected by the varying types and amounts of β-glucans present in the three diets.

Despite the marginal effect on the gut microbiota composition, the concentrations of total SCFA and acetic acid – the predominant individual SCFA – in cecal digesta were lower in broilers fed the high dose of fruiting bodies than in the other two broiler groups, and the concentration of propionic acid was lower in both groups fed the fruiting bodies than in the group fed no fruiting bodies. This indicates that microbial fermentation activity in the cecum was reduced by feeding the *P. sapidus* fruiting bodies. One explanation for this could be the absence and the low amount of wheat bran in the diets containing the fruiting bodies, respectively. As explained above, the partial or complete replacement of wheat bran in the diets containing the fruiting bodies was necessary to offset the high fiber content of the fruiting bodies and to make the CF content comparable across the three diets. However, CF analysis of the diets revealed that CF content was slightly higher in diet containing no fruiting bodies than in the other two diets. Thus, it is possible that the reduced fermentation activity seen in the broilers fed the high dose of fruiting bodies was caused by a lower provision of indigestible carbohydrates which serve as fermentation substrates. We also cannot exclude that phytate from wheat bran might have affected bacterial fermentation in the cecum of the broilers, because phytate is known to form complexes with different compounds in the diets and thus may affect substrate availability for fermentation (Cowieson et al., 2009).

A further factor that should be taken into account is that the high temperatures associated with pelleting were reported to affect the solubility of β-glucans in the ileal digesta of pigs fed barley-based diets due to disruption of the barley endosperm cell wall and, thereby, to increase the ileal digestibility of starch (Graham et al., 1989). This is explained by the fact that disruption of the endosperm cell wall increases the proportion of starch digested prior to the large intestine. As a consequence, the amount of starch for bacterial fermentation in the large intestine is reduced. The three diets in the present study differed in the amounts of several diet components (maize, wheat, wheat bran, fruiting bodies), and it is well known that the structures and physicochemical properties of β-glucans from different feed sources (cereals vs. fungi) markedly vary (Kaur et al., 2019; Choi and Kim, 2023). With regard to differences between cereal and fungal β-glucans, a substantially higher release of β-glucans and a stronger viscosity-inducing effect was observed after simulated digestion of oat and barley when compared to fungal samples (Colosimo et al., 2021a), - effects which have been attributed to the different β-linkages between plant and fungal polysaccharides and the different cell wall organisation/composition (Colosimo et al., 2021b). Thus, it is possible that differences in the release and solubility of β-glucans derived from the different feed components affected ileal digesta viscosity and substrate availability for bacterial fermentation in the cecum.

An analysis of gut permeability parameters, including plasma LPS concentration and the expression of genes involved in gut integrity - such as tight-junction proteins (*CLDN3, CLDN5, OCLN*) and mucin glycoproteins (*MUC2, MUC5AC, MUC13*) - in the cecum mucosa revealed no negative effects of including *P. sapidus* fruiting bodies in broiler diets. Both, tight-junction proteins and mucin glycoproteins are critical components of the gut barrier, and their decreased expression is often associated with gut barrier disruption and increased gut permeability (Ringseis et al., 2020). In contrast, mRNA levels of *CLDN3, MUC2*, and *MUC5AC* in the cecum mucosa were even higher in broilers fed the higher dose of *P. sapidus* fruiting bodies compared to those fed no fruiting bodies. However, while this stimulatory effect on the expression of gut barrier-protective genes could be considered beneficial, an improvement in gut barrier function due to dietary inclusion of *P. sapidus* fruiting bodies is unlikely, given that plasma LPS concentration did not differ among groups. Additionally, no differences were observed in the expression of several proinflammatory genes in the cecum mucosa among groups. This indicates that feeding *P. sapidus* fruiting bodies did not act as a detrimental stimulus to intestinal epithelial cells, which would typically provoke an inflammatory response (Ringseis et al., 2020). These findings are consistent with our recent study, which showed that feeding the vegetative mycelium of *P. sapidus* has no negative effects on gut integrity in broilers (Schäfer et al., 2024a). While the broilers of the present study were healthy and gut integrity was probably normal in these animals, it is possible that broilers under certain challenge conditions, such as presence of pathogens or heat stress, would benefit from the observed increase in the expression of gut barrier-protective genes, because both pathogens and heat stress are well-known to impair gut integrity and barrier function. In line with this, different indigestible carbohydrates were proven to be effective in protecting broilers from heat stress-induced impairments of gut morphology and function (Ringseis and Eder, 2022). Although increased cecal permeability in broilers has been demonstrated in response to feed restriction-induced leaky gut (Kuttappan et al., 2015; Vicuña et al., 2015), highlighting the cecum’s relevance for assessing intestinal integrity, investigations of gut integrity are typically focused on the small intestine. Therefore, future studies should also examine the potential impact of dietary fruiting bodies on gut integrity parameters in the small intestine.

Feeding of fungal biomass might affect intermediary metabolism through regulating gene expression in key metabolic tissues, because microbial products from fiber fermentation, such as SCFA, were shown to stimulate multiple SCFA receptors in these tissues and thereby modulate gene expression (Ringseis et al., 2020). To evaluate an impact of dietary inclusion of *P. sapidus* fruiting bodies on the intermediary metabolism of broilers, genome-wide transcriptome analysis of the liver and measurements of triglycerides and cholesterol in the liver were conducted. Despite the low cut-off filter setting for identifying differentially expressed genes, only 66 transcripts were identified in the group fed the higher dose and 47 transcripts in the group fed the lower dose of fruiting bodies compared to the group fed no *P. sapidus* fruiting bodies. Considering that only one gene (*PCK1*: FC = −2.07) was regulated > 2 or < −2 in the group fed the higher dose of fruiting bodies compared to the group fed no fruiting bodies, the effects of including *P. sapidus* fruiting bodies in the broiler diet on hepatic intermediary metabolism can be considered marginal. Against this background, the results of GSEA, which aim to interpret the biological significance of transcriptome-level changes, should be viewed with caution. According to the GSEA results, the most enriched GO term linked to upregulated genes pertained to potassium channel regulator activity. These genes were potassium voltage-gated channel interacting protein 4 (*KCNIP4*) and tsukushi (*TSKU*). In this context, it is particularly noteworthy that *KCNIP4* has been identified as an important candidate gene for rapid growth in chickens in a Chinese local broiler breed, according to a recent genome-wide association study (Wang et al., 2022). However, the potential biological mechanisms underlying the association between *KCNIP4* expression and growth remain to be elucidated. *TSKU* encodes a hepatokine that is produced and secreted by the liver and regulates metabolic and inflammatory conditions in the liver or at distant organs through systemic circulation (Meex and Watt, 2017). A recent study identified TSKU protein as a potential blood biomarker for non-alcoholic fatty liver disease (Mouchiroud et al., 2019). Furthermore, hepatic expression of *TSKU* was found to be strongly induced by stimuli that enhance thermogenesis and energy expenditure, such as T3 and cold exposure. Evidence suggests that the TSKU protein plays a critical role in the regulation of adipose tissue and systemic energy metabolism (Wang et al., 2019). In contrast, GSEA using the downregulated genes identified protein localization to the plasma membrane, glycerol metabolic process, and neurotransmitter receptor transport as the most enriched GO terms. The genes assigned to these terms included phosphoenolpyruvate carboxykinase 1 (*PCK1*), a well-known gluconeogenic key gene; the lipogenic gene monoacylglycerol O-acyltransferase 1 (*MOGAT1*); and the less well-characterized genes leucine-rich repeat-containing 7 (*LRRC7*), neuron-specific gene family member 1 (*NSG1*), and plakophilin 2 (*PKP2*). Despite the observed regulation of the *MOGAT1* gene, the unchanged concentrations of triglycerides and cholesterol in the liver of broilers across the groups suggest that feeding *P. sapidus* fruiting bodies does not have a profound effect on hepatic lipid metabolism. Therefore, *P. sapidus* fruiting bodies and *P. sapidus* mycelium appear similar in terms of their limited impact on the intermediary metabolism of broilers.

To determine whether the reduced breast weights observed in broilers fed the higher dose of fruiting bodies were due to an impairment of protein anabolic pathways or an activation of protein catabolic pathways, we analyzed the expression of several key genes associated with these pathways in breast muscle. However, the mRNA levels of none of the genes involved in either protein anabolic pathways (e.g., mTOR pathway, myogenesis) or protein catabolic pathways (e.g., GCN/eIF2a pathway, ubiquitin-proteasome pathway, autophagy-lysosomal pathway) differed among groups. Additionally, the mRNA levels of several proinflammatory genes (*IL8L1, TLR4, TNFA, VCAM1*) in breast muscle were similar across groups, suggesting that the muscle tissue did not experience a proinflammatory condition. Such a condition typically induces the expression of protein catabolic genes, such as *FBXO32* (atrogin-1) and *MURF1*, leading to muscle atrophy (Ramírez et al., 2011). Therefore, it is unlikely that the reduced breast weights—and presumably also the reduced thigh weights—in broilers fed the higher dose of fruiting bodies were caused by effects on muscle protein turnover.

As already discussed, a limitation of the study is that the inclusion of the fruiting bodies in the diets was accompanied by changes in the amounts of different diet components including maize, wheat and wheat bran. Owing to this, the effects observed cannot be solely ascribed to the feeding of fruiting bodies and its constituents. Another limitation of the study is the small number of broilers (24/group) and cages (6/group) used. Given that the cage constituted the observational unit for the performance data, the results should be regarded somewhat cautiously and validated in future studies involving a greater number of observations.

In conclusion, the present study demonstrates that the inclusion of *P. sapidus* fruiting bodies in broiler diets at 25 and 50 g/kg has marginal effects on cecal microbiota composition and intermediary metabolism in the liver and muscle but significantly impairs growth performance and the weights of eviscerated carcass and key meat cuts (breast, thigh). Based on these findings, the use of *P. sapidus* fruiting bodies as a dietary component for broilers cannot be recommended. Considering that dietary inclusion of *P. sapidus* mycelium, which has a significantly lower β-glucan content than the fruiting bodies, at identical doses did not impair growth performance or muscle weights in a recent broiler study (Schäfer et al., 2024b), it is plausible that the negative effects of the fruiting bodies on broiler performance are associated with their relatively high β-glucan content.

## Disclosures

The authors declare that they have no competing interests.

## Appendix Supplementary materials

Image, application 1
