# Supplementary material for: Influence of Pleurotus sapidus fruiting bodies on the performance, cecal microbiome, and gene expression in the liver and breast muscle of broilers
Source: Poult Sci. 2025 Jul 3;104(10):105517. doi: 10.1016/j.psj.2025.105517 (PMC12272587; doi:10.1016/j.psj.2025.105517)
Supplement: Supplementary file 1 [file mmc1.docx]

**Supplemental Table S1** Characteristics of *Gallus gallus* gene-specific primers used for qPCR analysis in liver, cecum mucosa and breast muscle of broilers fed diets with either 0 g (PSA-F0), 25 g (PSA-F25) or 50 g (PSA-F50) *P. sapidus* fruiting bodies per kg diet for 35 days.

| Gene symbol | Forward (5` to 3`), Reverse (5` to 3`) | PCR product size (bp) | NCBI GenBank accession no. |
| --- | --- | --- | --- |
| *Reference genes* |  |  |  |
| *ACTB* | ATGAAGCCCAGAGCAAAAGA, GGGGTGTTGAAGGTCTCAAA | 223 | NM205518 |
| *GAPDH* | ACTGTCAAGGCTGAGAACGG, AGCTGAGGGAGCTGAGATGA | 204 | NM204305 |
| *SDHA* | ATTCCCGTTTTGCCTACGGT**,** GGGAGTTTGCTCCAAGACGA | 172 | NM001277398 |
| *YWHAZ* | TTCCAACTTCCGTCTGCCTC**,** AGCAGTCTTCCTCGCTTGAC | 328 | NM001031343 |
| *Target genes* |  |  |  |
| *CLDN3* | TTTCGGTCAGCGGGTTCCTC, CCGTCACGATGTTGTTGCCG | 171 | NM204202 |
| *CLDN5* | ACTCATTGCAGGTCGCCAGA, AGCATCACGAGCGAGGGAAA | 147 | NM204201 |
| *4EBP1* | GCGAATGTAGGTGAAGAAGAGC, GGCTGGTGGGAATCCTCAAA | 108 | XM424384 |
| *ABCG2* | TGGGATGGAAGTGGTGGCTG, AAGGCCGTATCGAGGGATGC | 148 | NM001397256 |
| *ATG5* | GGCACCGACCGATTTAGT, GCTGATGGGTTTGCTTTT | 167 | NM001006409 |
| *ATG9A* | CCGCTATTGAGACAAGGGACGAG, CCGCAGGCAGATGATGAGGA | 115 | AM085507 |
| *BECN1* | CGACTGGAGCAGGAAGAAG, CGACTGGAGCAGGAAGAAG | 115 | NM001006332 |
| *COQ10B* | CCTTTGCAAGTGCCTGTGCT, ACCCGGAGCGCTTCGATAAT | 211 | NM001389593 |
| *CYP2W1* | CGCTCTTTGCTCCCATGCTG, AGCACTGGTGTGTCTTGGCT | 214 | XM003642141 |
| *FBXO32* | TGCCAGCTACAAGGCCGCAG, TGCTTGGCCAACGGAGGGGA | 296 | NM001030956 |
| *FOXO1* | CATAACCAGCCAACACCTGC, AATTCCCACCCTTCCGTAGC | 198 | NM204328 |
| *GPR146* | CTCAGCTCCAATCCCTCCTGG, GTGCTCCTCCACCATCCTCC | 95 | NM001277532 |
| *ICK* | GTTGGCAGCACTCTTGTAGCC, TGCTGCTTGTCCAGCTCTCA | 161 | XM004940476 |
| *IL1B* | TCTGCCTGCAGAAGAAGCCT, CGCTGTCAGCAAAGTCCCTG | 168 | NM204524 |
| *IL8L1* | GTGACACCCGGAAGAAACACT, CGTGCCTGAGCCATACCTTT | 109 | NM205018 |
| *IL8L2* | AGGCACTTATGGCCAAGGCT, GGCACCGATGTGAAAGGTGG | 157 | NM205498 |
| *LRRC7* | CTGAGGACGTTGGCTGTGGA, AGCGCAGAGACATCACGGTT | 91 | XM046923448 |
| *MOGAT1* | GCCAAGCACTGCCACAGAAG, ATCACACGGACACAGCTCCC | 146 | XM040679110 |
| *MTOR* | AGTGAGAGTGATGCGGAGAG, GAAACCTTGGACAGCGGG | 120 | XM417614.4 |
| *MUC13* | GCATTCCTCAAGCAGAGGTG, CTCAGGCTGCCGTGATATTT | 73 | XM015289971 |
| *MUC2* | ATTCCTTGTGACGCTGGACC, GTGTGGGAGCAGTGGTTGAT | 190 | NM001318434 |
| *MUC5AC* | ACATCACCCACAACACCGTT, TCTCCACCATCAAATCCAGGG | 158 | ENSGALG0000003414 |
| *MURF1* | GAACCTGCTGGTGGAGAACA, GTGCTCCCCCTTCTTGAGTG | 76 | XM424369 |
| *MYF5* | ACTCCCCAAAGTGGAGATCCT, ACTCCCCAAAGTGGAGATCCT | 154 | NM001030363 |
| *MYOD1* | AGGAAACCTGAGTGACAGTGGA, GCTTGGCTGAACGGAGCAA | 164 | NM204214 |
| *MYOG* | CGGGGTGGGATGGTGATG, TGGAGAGGAGTGGGAAAGGA | 112 | D90157 |
| *OCLN* | CGACAGCATCACCGAGGACA, TTTGGTAGTCTGGGCTCCGC | 92 | NM205128 |
| *PDK4* | TTGAGAGCTGTGATGGGCACT, CAATCTCCCACTGGCACTCCT | 191 | NM001199909 |
| *PLK4* | CAGGTGTAGCTCTGCACGGA, CCGCTGGTACTGATGCTGGA | 114 | XM040671402 |
| *PRSS35* | TCCACTATTCAGCACTGTGGC, CCGTTCCATTAGCAAGACCCA | 112 | XM040667415 |
| *RBPMS2* | TGGGCTCCCTACCCACTGTA, TCCTTGCTGGGTAGCTTCGG | 144 | NM204869 |
| *RHCE* | GGTGCGCAATGCCTTCTACC, ATCAGGAGGCTGAATCCGGTG | 232 | NM204467 |
| *RUNX2* | TAGGGCGCATTCCTCATCCC, TGGAGTGAATGGACGGCGAA | 207 | NM204128 |
| *S6K1* | GGTGGAGTTTGGGGGCATTA, GAAGAACGGGTGAGCCTGAA | 230 | NM001030721 |
| *SQSTM1* | CTGCGAGGGTAAAGGCATCC, CAGGGGAAGGGTGGAACAC | 132 | XM001233248 |
| *TCFL5* | AGTCGATGCTCCGACAGCTC, ATGGTCGGGATGGTGCTCAG | 131 | XM001234726 |
| *TLR4* | GTCCGTGGCTGGAGGTCATC, AGGTCCAAGTCCAGGGTGGT | 103 | NM001030693 |
| *TNFA* | TCGTGGCATCGTCCTCTCAG, GGTACTCCTCGGAGAAGCGG | 213 | MF000729 |
| *TOMM6* | GGATCGGTCGTGTCGTCTCC, CATCAGGTCGATGTCCGCCA | 183 | NM001302134 |
| *VCAM1* | GGAGAAACCGCCACTGTCAT, GGCTCAAAGTCCATACCAGCA | 89 | XM004936551 |
| *YPEL5* | CTGGATCACATTGGCGGCAC, GCCCAGTTTGCTGTTGCAGT | 228 | NM001007900 |
| *ZARL1* | CCCTGGAACCCTCAGCAACA, CTGCGAGCCGGTGCTAATTG | 94 | NM001171543 |

**Supplemental Table S2** Chemical composition of *P. sapidus* fruiting bodies

| Item | Concentration |
| --- | --- |
| *Analyzed crude nutrient and energy content* |  |
| Dry matter (% FM*) | 92.24 |
| CP (% DM) | 22.36 |
| EE (% DM) | 2.93 |
| CA (% DM) | 5.56 |
| CF (% DM) | 13.26 |
| Chitin (% DM) | 6.12 |
| Sugar (% DM) | 7.26 |
| Total glucans (% DM) | 50.58 |
| α-glucans (% DM) | 3.02 |
| β-glucans (% DM) | 47.56 |
| Gross energy (MJ/kg) | 18.61 |
| *Calculated energy content* |  |
| Metabolizable energy (MJ/kg) | 5.83 |
| *Amino acids (g/kg)* |  |
| Alanine | 20.94 |
| Arginine | 9.64 |
| Asparagine/aspartic acid | 13.96 |
| Cysteine | 2.09 |
| Glutamine/glutamic acid | 25.59 |
| Glycine | 7.42 |
| Histidine | 3.75 |
| Isoleucine | 6.59 |
| Leucine | 11.83 |
| Lysine | 9.21 |
| Methionine | 4.13 |
| Phenylalanine | 7.26 |
| Proline | 7.52 |
| Serine | 8.64 |
| Threonine | 7.81 |
| Tryptophan | 3.04 |
| Tyrosine | 5.35 |
| Valine | 7.50 |
| Fatty acids, % of total fatty acids^#^ |  |
| C14:0 | 0.277 |
| C16:0 | 17.68 |
| C18:0 | 5.28 |
| C18:1 | 20.37 |
| C18:2 | 56.01 |

*FM, fresh matter after freeze drying; Abbreviations: FM, fresh matter; DM, dry matter; CP, crude protein; EE, ether extract; CA, crude ash; CF, crude fiber

^#^Only fatty acids > 0.1 % of total fatty acids are shown.

**Supplemental Table S3** Relative abundance of bacterial taxa in cecum digesta of broilers fed diets with either 0 g (PSA-F0), 25 g (PSA-F25) or 50 g (PSA-F50) *P. sapidus* fruiting bodies per kg diet for 35 days.

|  | PSA-F0 | PSA-F25 | PSA-F50 | *P-*value |
| --- | --- | --- | --- | --- |
| Phylum |  |  |  |  |
| Actinobacteriota | 0.12 ± 0.05 | 0.09 ± 0.02 | 0.10 ± 0.05 | 0.232 |
| Firmicutes | 96.3 ± 2.2 | 97.0 ± 2.0 | 94.4 ± 3.6 | 0.105 |
| Proteobacteria | 3.60 ± 2.20 | 2.92 ± 2.01 | 5.31 ± 3.43 | 0.105 |
| Verrucomicrobiota | 0.01 ± 0.01 | 0.01 ± 0.01 | 0.16 ± 0.33 | 0.232 |
| Class |  |  |  |  |
| Bacilli | 12.4 ± 7.6^b^ | 21.6 ± 12.6^ab^ | 24.3 ± 9.8^a^ | 0.025 |
| Clostridia | 83.9 ± 7.8^a^ | 75.3 ± 12.6^ab^ | 70.1 ± 7.7^b^ | 0.025 |
| Coriobacteriia | 0.12 ± 0.05 | 0.09 ± 0.02 | 0.10 ± 0.05 | 0.232 |
| Gammaproteobacteria | 3.60 ± 2.20 | 2.92 ± 2.01 | 5.31 ± 3.43 | 0.087 |
| Verrucomicrobiae | 0.01 ± 0.01 | 0.01 ± 0.01 | 0.16 ± 0.33 | 0.232 |
| Order |  |  |  |  |
| Burkholderiales | 0.19 ± 0.45 | 0.20 ± 0.36 | 0.61 ± 0.82 | 0.698 |
| Clostridia UCG 014 | 0.69 ± 1.08 | 0.49 ± 0.74 | 0.16 ± 0.24 | 0.726 |
| Clostridiales | 0.06 ± 0.10 | 0.33 ± 0.64 | 0.25 ± 0.55 | 0.726 |
| Coriobacteriales | 0.12 ± 0.05 | 0.09 ± 0.02 | 0.10 ± 0.05 | 0.395 |
| Enterobacterales | 3.41 ± 2.16 | 2.72 ± 2.13 | 4.70 ± 3.30 | 0.276 |
| Erysipelotrichales | 2.26 ± 1.25 | 2.09 ± 1.08 | 1.98 ± 1.24 | 0.726 |
| Eubacteriales | 0.01 ± 0.01 | 0.01 ± 0.01 | 0.01 ± 0.01 | 0.276 |
| Lachnospirales | 68.7 ± 8.6 | 63.1 ± 11.5 | 58.9 ± 5.1 | 0.198 |
| Lactobacillales | 10.1 ± 8.2 | 19.6 ± 13.6 | 22.3 ± 10.5 | 0.198 |
| Monoglobales | 0.84 ± 0.69 | 0.46 ± 0.32 | 0.50 ± 0.27 | 0.276 |
| Oscillospirales | 6.15 ± 1.52 | 7.33 ± 2.18 | 5.81 ± 2.47 | 0.726 |
| Peptococcales | 0.18 ± 0.18 | 0.11 ± 0.08 | 0.13 ± 0.13 | 0.726 |
| Peptostreptococcales Tissierellales | 7.32 ± 5.48 | 3.53 ± 1.98 | 4.33 ± 3.45 | 0.395 |
| Verrucomicrobiales | 0.01 ± 0.01 | 0.01 ± 0.01 | 0.16 ± 0.33 | 0.405 |
| Family |  |  |  |  |
| Anaerofustaceae | 0.01 ± 0.01 | 0.01 ± 0.01 | 0.01 ± 0.01 | 0.361 |
| Akkermansiaceae | 0.01 ± 0.01 | 0.01 ± 0.01 | 0.16 ± 0.33 | 0.566 |
| Clostridium methylpentosum group | 0.01 ± 0.01 | 0.01 ± 0.01 | 0.01 ± 0.01 | 0.842 |
| Morganellaceae | 0.01 ± 0.02 | 0.03 ± 0.04 | 0.11 ± 0.22 | 0.204 |
| Eggerthellaceae | 0.12 ± 0.05 | 0.09 ± 0.02 | 0.10 ± 0.05 | 0.543 |
| Oscillospirales | 0.07 ± 0.09 | 0.10 ± 0.16 | 0.10 ± 0.13 | 0.853 |
| Peptococcaceae | 0.18 ± 0.18 | 0.11 ± 0.08 | 0.13 ± 0.13 | 0.842 |
| Sutterellaceae | 0.19 ± 0.45 | 0.20 ± 0.36 | 0.61 ± 0.82 | 0.842 |
| Clostridiaceae | 0.06 ± 0.10 | 0.33 ± 0.64 | 0.25 ± 0.55 | 0.842 |
| Monoglobaceae | 0.84 ± 0.69 | 0.46 ± 0.32 | 0.50 ± 0.27 | 0.361 |
| Clostridia UCG 014 | 0.69 ± 1.08 | 0.49 ± 0.74 | 0.16 ± 0.24 | 0.842 |
| Eubacterium coprostanoligenes group | 0.40 ± 0.21 | 0.53 ± 0.27 | 0.57 ± 0.45 | 0.842 |
| Erysipelotrichaceae | 1.30 ± 1.10 | 0.88 ± 0.48 | 0.92 ± 0.78 | 0.842 |
| Erysipelatoclostridiaceae | 0.97 ± 0.50 | 1.22 ± 0.72 | 1.06 ± 0.64 | 0.853 |
| Enterococcaceae | 1.59 ± 2.09 | 1.32 ± 2.41 | 1.73 ± 2.57 | 0.853 |
| Oscillospiraceae | 1.21 ± 0.66 | 1.64 ± 0.88 | 1.16 ± 0.70 | 0.842 |
| Butyricicoccaceae | 2.08 ± 0.45 | 2.31 ± 0.6 | 1.97 ± 0.53 | 0.842 |
| Enterobacteriaceae | 3.40 ± 2.20 | 2.69 ± 2.13 | 4.59 ± 3.19 | 0.361 |
| Ruminococcaceae | 2.38 ± 0.82 | 2.74 ± 1.20 | 2.00 ± 1.00 | 0.842 |
| Peptostreptococcaceae | 7.32 ± 5.48 | 3.53 ± 1.98 | 4.33 ± 3.45 | 0.543 |
| Lactobacillaceae | 8.51 ± 7.83 | 18.2 ± 14.5 | 20.6 ± 12.3 | 0.207 |
| Lachnospiraceae | 68.7 ± 8.6 | 63.1 ± 11.5 | 58.9 ± 5.1 | 0.207 |
| Genus |  |  |  |  |
| *Akkermansia* | 0.01 ± 0.01 | 0.01 ± 0.01 | 0.16 ± 0.33 | 0.544 |
| *Anaerofustis* | 0.01 ± 0.01 | 0.01 ± 0.01 | 0.01 ± 0.01 | 0.443 |
| *Anaerostipes* | 2.91 ± 1.82 | 5.12 ± 3.82 | 3.77 ± 1.79 | 0.538 |
| *Anaerotruncus* | 0.25 ± 0.18 | 0.18 ± 0.15 | 0.26 ± 0.18 | 0.592 |
| *ASF356* | 0.14 ± 0.11 | 0.09 ± 0.07 | 0.10 ± 0.10 | 0.739 |
| *Blautia* | 6.18 ± 3.27 | 7.54 ± 5.4 | 5.21 ± 2.75 | 0.739 |
| *Butyricicoccus* | 1.95 ± 0.43 | 2.14 ± 0.53 | 1.83 ± 0.55 | 0.875 |
| *Caproiciproducens* | 0.04 ± 0.03 | 0.07 ± 0.08 | 0.03 ± 0.02 | 0.443 |
| *CHKCI001* | 0.27 ± 0.20 | 0.32 ± 0.20 | 0.56 ± 0.31 | 0.263 |
| *Clostridia UCG 014* | 0.69 ± 1.08 | 0.489 ± 0.74 | 0.16 ± 0.24 | 0.799 |
| *Clostridioides* | 0.01 ± 0.01 | 0.01 ± 0.01 | 0.06 ± 0.11 | 0.366 |
| *Clostridium innocuum group* | 0.42 ± 1.17 | 0.16 ± 0.24 | 0.19 ± 0.21 | 0.875 |
| *Clostridium methylpentosum group* | 0.01 ± 0.01 | 0.01 ± 0.01 | 0.01 ± 0.01 | 0.739 |
| *Clostridium sensu stricto 1* | 0.06 ± 0.10 | 0.33 ± 0.64 | 0.25 ± 0.55 | 0.838 |
| *Colidextribacter* | 0.43 ± 0.27 | 0.89 ± 0.54 | 0.45 ± 0.30 | 0.544 |
| *DTU089* | 0.29 ± 0.27 | 0.45 ± 0.17 | 0.42 ± 0.42 | 0.538 |
| *Eggerthella* | 0.08 ± 0.04 | 0.05 ± 0.02 | 0.06 ± 0.04 | 0.587 |
| *Eisenbergiella* | 5.74 ± 2.96 | 5.76 ± 1.33 | 6.26 ± 2.47 | 0.592 |
| *Enterococcus* | 1.59 ± 2.09 | 1.32 ± 2.41 | 1.73 ± 2.57 | 0.875 |
| *Erysipelatoclostridium* | 0.97 ± 0.50 | 1.22 ± 0.72 | 1.06 ± 0.64 | 0.875 |
| *Escherichia Shigella* | 3.38 ± 2.17 | 2.68 ± 2.12 | 4.51 ± 3.13 | 0.443 |
| *Eubacterium coprostanoligenes group* | 0.40 ± 0.21 | 0.53 ± 0.27 | 0.57 ± 0.45 | 0.838 |
| *Eubacterium hallii group* | 0.85 ± 0.58 | 0.69 ± 0.37 | 0.66 ± 0.43 | 0.838 |
| *Flavonifractor* | 0.23 ± 0.11 | 0.20 ± 0.13 | 0.27 ± 0.20 | 0.799 |
| *GCA 900066575* | 1.23 ± 2.50 | 0.57 ± 0.81 | 1.09 ± 1.20 | 0.544 |
| *Gordonibacter* | 0.05 ± 0.02 | 0.04 ± 0.02 | 0.04 ± 0.01 | 0.653 |
| *Hydrogenoanaerobacterium* | 0.07 ± 0.09 | 0.10 ± 0.16 | 0.10 ± 0.13 | 0.875 |
| *Incertae Sedis* | 0.64 ± 0.47 | 0.93 ± 0.35 | 0.59 ± 0.28 | 0.538 |
| *Klebsiella* | 0.03 ± 0.07 | 0.01 ± 0.02 | 0.08 ± 0.09 | 0.209 |
| *Lachnoclostridium* | 0.77 ± 0.49 | 0.93 ± 0.38 | 0.91 ± 0.95 | 0.739 |
| *Lachnospiraceae UCG 010* | 0.01 ± 0.01 | 0.01 ± 0.01 | 0.03 ± 0.07 | 0.739 |
| *Lactobacillus* | 6.17 ± 5.01 | 13.8 ± 14.1 | 12.5 ± 12.2 | 0.592 |
| *Monoglobus* | 0.84 ± 0.69 | 0.46 ± 0.32 | 0.50 ± 0.27 | 0.443 |
| *Oscillibacter* | 0.31 ± 0.39 | 0.25 ± 0.28 | 0.19 ± 0.18 | 0.875 |
| *Paludicola* | 0.02 ± 0.03 | 0.03 ± 0.06 | 0.03 ± 0.03 | 0.653 |
| *Parasutterella* | 0.19 ± 0.45 | 0.20 ± 0.36 | 0.61 ± 0.82 | 0.739 |
| *Pediococcus* | 2.34 ± 4.49 | 4.46 ± 5.12 | 8.10 ± 6.60 | 0.209 |
| *Proteus* | 0.01 ± 0.02 | 0.03 ± 0.04 | 0.11 ± 0.22 | 0.209 |
| *Romboutsia* | 7.16 ± 5.41 | 3.42 ± 1.82 | 4.09 ± 3.17 | 0.544 |
| *Ruminococcus torques group* | 14.1 ± 5.9 | 9.76 ± 2.98 | 11.0 ± 5.0 | 0.523 |
| *Sellimonas* | 4.23 ± 1.89 | 3.89 ± 1.24 | 3.64 ± 1.15 | 0.875 |
| *Shuttleworthia* | 0.63 ± 0.59 | 0.24 ± 0.25 | 0.38 ± 0.31 | 0.523 |
| *Turicibacter* | 0.32 ± 0.41 | 0.03 ± 0.05 | 0.27 ± 0.77 | 0.443 |
| *Tyzzerella* | 0.11 ± 0.05 | 0.09 ± 0.03 | 0.07 ± 0.03 | 0.544 |
| *UCG 009* | 0.13 ± 0.07 | 0.17 ± 0.11 | 0.14 ± 0.07 | 0.875 |
| *uncultured Erysipelotrichaceae* | 0.56 ± 0.46 | 0.69 ± 0.52 | 0.47 ± 0.38 | 0.875 |
| *uncultured Lachnospiraceae* | 1.10 ± 0.60 | 0.98 ± 0.71 | 1.04 ± 0.75 | 0.875 |
| *uncultured Oscillospiraceae* | 0.19 ± 0.06 | 0.23 ± 0.05 | 0.19 ± 0.06 | 0.739 |
| *uncultured Peptococcaceae* | 0.18 ± 0.18 | 0.11 ± 0.08 | 0.13 ± 0.13 | 0.875 |
| *uncultured Ruminococcaceae* | 0.31 ± 0.10 | 0.35 ± 0.16 | 0.21 ± 0.10 | 0.263 |
| *unknown Lachnospiraceae* | 30.4 ± 9.6 | 27.1 ± 5.8 | 24.3 ± 6.7 | 0.544 |
| *unknown Oscillospiraceae* | 0.05 ± 0.05 | 0.06 ± 0.07 | 0.07 ± 0.09 | 0.896 |
| *unknown Peptostreptococcaceae* | 0.16 ± 0.34 | 0.10 ± 0.30 | 0.18 ± 0.54 | 0.210 |
| *unknown Ruminococcaceae* | 0.82 ± 0.68 | 0.73 ± 0.82 | 0.48 ± 0.44 | 0.739 |
| Species |  |  |  |  |
| *Anaerofustis stercorihominis* | 0.01 ± 0.01 | 0.01 ± 0.01 | 0.01 ± 0.01 | 0.437 |
| *Anaerostipes butyraticus* | 2.82 ± 1.82 | 5.03 ± 3.86 | 3.67 ± 1.87 | 0.639 |
| *Anaerostipes caccae* | 0.01 ± 0.01 | 0.01 ± 0.01 | 0.01 ± 0.01 | 0.742 |
| *bacterium ic1311* | 1.10 ± 0.60 | 0.98 ± 0.71 | 1.04 ± 0.75 | 0.919 |
| *Blautia hydrogenotrophica* | 0.33 ± 0.17 | 0.37 ± 0.21 | 0.48 ± 0.39 | 0.919 |
| *Clostridiales bacterium* | 0.56 ± 0.46 | 0.69 ± 0.52 | 0.47 ± 0.38 | 0.919 |
| *Clostridioides difficile* | 0.01 ± 0.01 | 0.01 ± 0.01 | 0.06 ± 0.11 | 0.429 |
| *Clostridium methylpentosum* | 0.01 ± 0.01 | 0.01 ± 0.01 | 0.01 ± 0.01 | 0.746 |
| *Clostridium paraputrificum* | 0.01 ± 0.01 | 0.01 ± 0.01 | 0.02 ± 0.02 | 0.429 |
| *Clostridium perfringens* | 0.06 ± 0.09 | 0.33 ± 0.64 | 0.23 ± 0.55 | 0.989 |
| *Gordonibacter pamelaeae* | 0.05 ± 0.02 | 0.04 ± 0.02 | 0.04 ± 0.01 | 0.692 |
| *Lachnoclostridium phocaeense* | 1.27 ± 0.53 | 1.20 ± 0.50 | 1.04 ± 0.32 | 0.746 |
| *Lactobacillus gasseri* | 6.17 ± 5.01 | 13.8 ± 14.1 | 12.5 ± 12.2 | 0.657 |
| *Massiliomicrobiota timonensis* | 0.94 ± 0.49 | 1.13 ± 0.7 | 0.88 ± 0.64 | 0.919 |
| *metagenome* | 0.18 ± 0.07 | 0.19 ± 0.13 | 0.13 ± 0.10 | 0.675 |
| *uncultured bacterium ASF356* | 0.14 ± 0.11 | 0.09 ± 0.07 | 0.10 ± 0.10 | 0.742 |
| *uncultured bacterium Butyricicoccus* | 0.16 ± 0.15 | 0.17 ± 0.09 | 0.07 ± 0.05 | 0.429 |
| *uncultured bacterium Caproiciproducens* | 0.04 ± 0.03 | 0.07 ± 0.08 | 0.03 ± 0.02 | 0.437 |
| *uncultured bacterium Eggerthella* | 0.08 ± 0.04 | 0.05 ± 0.02 | 0.06 ± 0.04 | 0.639 |
| *uncultured bacterium Eubacterium hallii group* | 0.85 ± 0.58 | 0.69 ± 0.37 | 0.66 ± 0.43 | 0.816 |
| *uncultured bacterium Flavonifractor* | 0.17 ± 0.11 | 0.14 ± 0.11 | 0.20 ± 0.10 | 0.746 |
| *uncultured bacterium Hydrogenoanaerobacterium* | 0.07 ± 0.09 | 0.10 ± 0.16 | 0.10 ± 0.13 | 0.919 |
| *uncultured bacterium Lachnospiraceae UCG 010* | 0.01 ± 0.01 | 0.01 ± 0.01 | 0.03 ± 0.07 | 0.742 |
| *uncultured bacterium Monoglobus* | 0.84 ± 0.69 | 0.46 ± 0.32 | 0.50 ± 0.27 | 0.429 |
| *uncultured bacterium Ruminococcus torques group* | 5.73 ± 1.35 | 4.95 ± 1.23 | 4.57 ± 0.80 | 0.429 |
| *uncultured bacterium Sellimonas* | 0.72 ± 0.42 | 1.32 ± 0.88 | 0.89 ± 0.88 | 0.498 |
| *uncultured bacterium Shuttleworthia* | 0.63 ± 0.59 | 0.24 ± 0.25 | 0.38 ± 0.31 | 0.535 |
| *uncultured bacterium Tyzzerella* | 0.11 ± 0.05 | 0.09 ± 0.03 | 0.07 ± 0.03 | 0.632 |
| *uncultured bacterium uncultured* | 0.18 ± 0.18 | 0.11 ± 0.08 | 0.13 ± 0.13 | 0.904 |
| *uncultured Clostridiales Ruminococcus torques group* | 8.32 ± 6.06 | 4.81 ± 2.57 | 6.38 ± 4.36 | 0.742 |
| *uncultured organism Akkermansia* | 0.01 ± 0.01 | 0.01 ± 0.01 | 0.16 ± 0.32 | 0.632 |
| *uncultured organism Anaerotruncus* | 0.25 ± 0.18 | 0.18 ± 0.15 | 0.26 ± 0.18 | 0.657 |
| *uncultured organism Butyricicoccus* | 0.56 ± 0.16 | 0.59 ± 0.17 | 0.53 ± 0.17 | 0.922 |
| *uncultured organism CHKCI001* | 0.27 ± 0.20 | 0.32 ± 0.20 | 0.56 ± 0.31 | 0.331 |
| *uncultured organism Incertae Sedis* | 0.36 ± 0.32 | 0.53 ± 0.29 | 0.29 ± 0.18 | 0.632 |
| *uncultured organism Lachnoclostridium* | 0.38 ± 0.30 | 0.33 ± 0.12 | 0.23 ± 0.11 | 0.639 |
| *uncultured organism Oscillibacter* | 0.30 ± 0.38 | 0.24 ± 0.26 | 0.17 ± 0.17 | 0.919 |
| *uncultured organism Parasutterella* | 0.13 ± 0.30 | 0.14 ± 0.25 | 0.42 ± 0.56 | 0.742 |
| *uncultured organism Sellimonas* | 0.10 ± 0.09 | 0.10 ± 0.07 | 0.12 ± 0.08 | 0.816 |
| *unknown Anaerostipes* | 0.08 ± 0.08 | 0.09 ± 0.10 | 0.10 ± 0.18 | 0.816 |
| *unknown Blautia* | 5.85 ± 3.36 | 7.17 ± 5.43 | 4.74 ± 2.83 | 0.742 |
| *unknown Butyricicoccus* | 1.25 ± 0.36 | 1.38 ± 0.36 | 1.23 ± 0.36 | 0.935 |
| *unknown Clostridia UCG 014* | 0.69 ± 1.08 | 0.49 ± 0.74 | 0.16 ± 0.24 | 0.803 |
| *unknown Clostridium innocuum group* | 0.42 ± 1.17 | 0.16 ± 0.24 | 0.19 ± 0.21 | 0.919 |
| *unknown Colidextribacter* | 0.43 ± 0.27 | 0.89 ± 0.54 | 0.45 ± 0.30 | 0.632 |
| *unknown DTU089* | 0.29 ± 0.27 | 0.45 ± 0.17 | 0.42 ± 0.42 | 0.620 |
| *unknown Eisenbergiella* | 5.74 ± 2.96 | 5.76 ± 1.33 | 6.26 ± 2.47 | 0.657 |
| *unknown Enterococcus* | 1.59 ± 2.09 | 1.32 ± 2.41 | 1.73 ± 2.57 | 0.919 |
| *unknown Erysipelatoclostridium* | 0.03 ± 0.02^c^ | 0.09 ± 0.04^b^ | 0.17 ± 0.06^a^ | 0.001 |
| *unknown Escherichia Shigella* | 3.38 ± 2.17 | 2.68 ± 2.12 | 4.51 ± 3.13 | 0.429 |
| *unknown Eubacterium coprostanoligenes group* | 0.40 ± 0.21 | 0.53 ± 0.27 | 0.57 ± 0.45 | 0.816 |
| *unknown Flavonifractor* | 0.06 ± 0.08 | 0.06 ± 0.05 | 0.07 ± 0.10 | 0.919 |
| *unknown GCA 900066575* | 1.23 ± 2.50 | 0.57 ± 0.81 | 1.09 ± 1.20 | 0.632 |
| *unknown Incertae Sedis* | 0.28 ± 0.17 | 0.40 ± 0.22 | 0.30 ± 0.10 | 0.792 |
| *unknown Klebsiella* | 0.03 ± 0.07 | 0.01 ± 0.02 | 0.08 ± 0.09 | 0.206 |
| *unknown Lachnoclostridium* | 0.39 ± 0.24 | 0.59 ± 0.40 | 0.68 ± 0.86 | 0.764 |
| *unknown Lachnospiraceae* | 30.4 ± 9.6 | 27.1 ± 5.8 | 24.3 ± 6.7 | 0.632 |
| *unknown Oscillibacter* | 0.01 ± 0.02 | 0.01 ± 0.02 | 0.01 ± 0.01 | 0.816 |
| *unknown Oscillospiraceae* | 0.05 ± 0.05 | 0.06 ± 0.07 | 0.07 ± 0.09 | 0.922 |
| *unknown Paludicola* | 0.02 ± 0.02 | 0.03 ± 0.06 | 0.03 ± 0.03 | 0.692 |
| *unknown Parasutterella* | 0.06 ± 0.14 | 0.06 ± 0.11 | 0.19 ± 0.25 | 0.742 |
| *unknown Pediococcus* | 2.34 ± 4.49 | 4.46 ± 5.12 | 8.10 ± 6.60 | 0.206 |
| *unknown Peptostreptococcaceae* | 0.16 ± 0.34 | 0.10 ± 0.30 | 0.18 ± 0.54 | 0.221 |
| *unknown Proteus* | 0.01 ± 0.02 | 0.03 ± 0.04 | 0.11 ± 0.22 | 0.206 |
| *unknown Romboutsia* | 7.16 ± 5.41 | 3.42 ± 1.82 | 4.09 ± 3.17 | 0.632 |
| *unknown Ruminococcaceae* | 0.82 ± 0.68 | 0.73 ± 0.82 | 0.48 ± 0.44 | 0.742 |
| *unknown Sellimonas* | 2.14 ± 1.56 | 1.26 ± 1.10 | 1.60 ± 0.80 | 0.675 |
| *unknown Turicibacter* | 0.32 ± 0.41 | 0.03 ± 0.05 | 0.27 ± 0.77 | 0.429 |
| *unknown UCG 009* | 0.13 ± 0.07 | 0.17 ± 0.11 | 0.14 ± 0.07 | 0.919 |
| *unknown uncultured Oscillospiraceae* | 0.19 ± 0.06 | 0.23 ± 0.05 | 0.19 ± 0.06 | 0.746 |
| *unknown uncultured Ruminococcaceae* | 0.14 ± 0.07 | 0.156 ± 0.09 | 0.08 ± 0.05 | 0.429 |

Data are means ± SD for *n* = 12 broilers/group. ^a,b,c^Means without a common letter differ across the groups.

**Supplemental Table S4** Liver weight and concentrations of triglycerides and cholesterol in liver and plasma of broilers fed diets with either 0 g (PSA-F0), 25 g (PSA-F25) or 50 g (PSA-F50) *P. sapidus* fruiting bodies per kg diet for 35 days.

|  | PSA-F0 | PSA-F25 | PSA-F50 | *P*-value |
| --- | --- | --- | --- | --- |
| Liver weight |  |  |  |  |
| Absolute (g) | 52.2 ± 4.6 | 50.7 ± 6.3 | 53.5 ± 4.4 | 0.409 |
| Relative (% of BW) | 1.76 ± 0.12 | 1.84 ± 0.17 | 1.83 ± 0.11 | 0.324 |
| Liver |  |  |  |  |
| Triglycerides (µmol/g) | 24.4 ± 7.8 | 26.6 ± 8.1 | 25.9 ± 4.4 | 0.805 |
| Cholesterol (µmol/g) | 10.4 ± 0.5 | 10.4 ± 0.4 | 10.2 ± 0.9 | 0.773 |
| Plasma |  |  |  |  |
| Triglycerides (mmol/l) | 1.04 ± 0.26 | 1.07 ± 0.37 | 1.31 ± 0.44 | 0.173 |
| Cholesterol (mmol/l) | 3.27 ± 0.34^a^ | 2.67 ± 0.81^b^ | 3.07 ± 0.43^ab^ | 0.045 |

Data are means ± SD, *n* = 12 broilers/group. ^a,b^Means without a common letter differ across the groups, *P* < 0.05. Abbreviation: BW, body weight.

**Supplemental Table S5** Relative mRNA levels of selected genes involved in protein synthesis and protein degradation in breast muscle of broilers fed diets with either 0 g (PSA-F0), 25 g (PSA-F25) or 50 g (PSA-F50) *P. sapidus* fruiting bodies per kg diet for 35 days.

|  | PSA-F0 | PSA-F25 | PSA-F50 | *P*-value |
| --- | --- | --- | --- | --- |
|  | Normalized mRNA level (fold of PSA-0) | | |  |
| Proinflammatory genes |  |  |  |  |
| *IL8L1* | 1.00 ± 0.86 | 1.58 ± 1.12 | 1.95 ± 1.19 | 0.067 |
| *TLR4* | 1.00 ± 0.89 | 0.87 ± 0.62 | 0.708 ± 0.27 | 0.931 |
| *TNFA* | 1.00 ± 0.42 | 0.86 ± 0.72 | 0.604 ± 0.38 | 0.105 |
| *VCAM1* | 1.00 ± 0.29 | 0.86 ± 0.35 | 0.848 ± 0.32 | 0.481 |
| mTOR pathway |  |  |  |  |
| *MTOR* | 1.00 ± 0.22 | 1.08 ± 0.44 | 0.90 ± 0.28 | 0.407 |
| *S6K1* | 1.00 ± 0.56 | 0.92 ± 0.53 | 0.86 ± 0.33 | 0.795 |
| *4EBP1* | 1.00 ± 0.16 | 1.03 ± 0.19 | 1.06 ± 0.20 | 0.761 |
| Muscle growth |  |  |  |  |
| *MYF5* | 1.00 ± 0.37 | 1.61 ± 0.79 | 1.23 ± 0.35 | 0.077 |
| *MYOD1* | 1.00 ± 0.14 | 0.99 ± 0.16 | 0.91 ± 0.12 | 0.229 |
| *MYOG* | 1.00 ± 0.44 | 1.04 ± 0.56 | 1.15 ± 0.19 | 0.215 |
| GCN/eIF2a pathway |  |  |  |  |
| *SQSTM1* | 1.00 ± 0.15 | 1.33 ± 0.47 | 1.03 ± 0.22 | 0.098 |
| Ubiquitin-proteasome pathway |  |  |  |  |
| *FBXO32* | 1.00 ± 0.35 | 1.37 ± 0.79 | 0.88 ± 0.48 | 0.228 |
| *FOXO1* | 1.00 ± 0.16 | 0.90 ± 0.14 | 0.84 ± 0.14 | 0.054 |
| *MURF1* | 1.00 ± 0.28 | 1.03 ± 0.24 | 0.92 ± 0.15 | 0.575 |
| Autophagy-lysosomal pathway |  |  |  |  |
| *ATG5* | 1.00 ± 0.14 | 1.1 ± 0.22 | 0.949 ± 0.07 | 0.105 |
| *ATG9A* | 1.00 ± 0.28 | 0.85 ± 0.21 | 0.878 ± 0.19 | 0.261 |
| *BECN1* | 1.00 ± 0.11 | 0.98 ± 0.15 | 0.92 ± 0.15 | 0.411 |

Data are means ± SD, *n* = 12 broilers/group. ^a,b^Means without a common letter differ across the groups, *P* < 0.05.

**Supplemental Table S6** Differentially expressed transcripts (filter criteria: FC > 1.3 and < -1.3 and *P* < 0.05) in the liver of broilers between groups PSA-F50 vs. PSA-F0

| Gene symbol | Gene description | PSA-F5 vs. PSA-F0 | | | PSA-F2.5 vs. PSA-F0 | | |
| --- | --- | --- | --- | --- | --- | --- | --- |
|  |  | SLR | FC | *P*-value | SLR | FC | *P*-value |
| *C12orf75* | Chromosome 12 open reading frame 75 | 0.73 | 1.65 | 0.006 | 0.20 | 1.15 | 0.279 |
| *COQ10B* | Coenzyme Q10B | 0.71 | 1.64 | 0.005 | 1.03 | 2.04 | 0.065 |
| *PDK4* | Pyruvate dehydrogenase kinase, isozyme 4 | 0.64 | 1.56 | 0.028 | 1.03 | 2.04 | 0.058 |
| *LOC101749797* | Uncharacterized LOC101749797 | 0.60 | 1.51 | 0.030 | 0.14 | 1.10 | 0.643 |
| *XDH* | Xanthine dehydrogenase | 0.58 | 1.50 | 0.023 | 0.91 | 1.87 | 0.052 |
| *ABCG2* | ATP-binding cassette, sub-family G (WHITE), member 2 (Junior blood group) | 0.57 | 1.49 | 0.010 | 0.48 | 1.40 | 0.046 |
| *CHST4* | Carbohydrate (N-acetylglucosamine 6-O) sulfotransferase 4 | 0.53 | 1.45 | 0.016 | -0.05 | -1.04 | 0.822 |
| *ATP6V0D2* | Atpase, H+ transporting, lysosomal 38kda, V0 subunit d2 | 0.50 | 1.41 | 0.045 | 0.17 | 1.12 | 0.351 |
| *TDO2* | Tryptophan 2,3-dioxygenase | 0.49 | 1.40 | 0.006 | 0.37 | 1.29 | 0.060 |
| *DHRS3* | Dehydrogenase/reductase (SDR family) member 3 | 0.48 | 1.40 | 0.010 | 0.62 | 1.53 | 0.008 |
| *PSD2* | Pleckstrin and Sec7 domain containing 2 | 0.46 | 1.38 | 0.020 | 0.22 | 1.16 | 0.183 |
| *SPATA13* | Spermatogenesis associated 13 | 0.45 | 1.37 | 0.014 | 0.26 | 1.20 | 0.059 |
| *SLC22A13L* | Solute carrier family 22 member 13-like | 0.45 | 1.37 | 0.024 | 0.38 | 1.30 | 0.140 |
| *BCMO1* | Beta-carotene 15,15-monooxygenase 1 | 0.45 | 1.36 | 0.016 | 0.35 | 1.28 | 0.077 |
| *RHPN2* | Rhophilin, Rho gtpase binding protein 2 | 0.44 | 1.36 | 0.024 | 0.18 | 1.13 | 0.261 |
| *FAM19A5* | Family with sequence similarity 19 (chemokine (C-C motif)-like), member A5 | 0.44 | 1.36 | 0.011 | 0.34 | 1.27 | 0.067 |
| *ICK* | Intestinal cell (MAK-like) kinase | 0.44 | 1.35 | 0.010 | 0.20 | 1.15 | 0.190 |
| *FAM3B* | Family with sequence similarity 3, member B | 0.43 | 1.35 | 0.008 | 0.33 | 1.26 | 0.030 |
| *PNAT10* | N-acetyltransferase, pineal gland isozyme NAT-10 | 0.43 | 1.35 | 0.013 | 0.30 | 1.23 | 0.101 |
| *KCNIP4* | Kv channel interacting protein 4 | 0.43 | 1.34 | 0.034 | 0.18 | 1.14 | 0.392 |
| *CHKA* | Choline kinase alpha | 0.42 | 1.34 | 0.043 | 0.03 | 1.02 | 0.890 |
| *YPEL5* | Yippee-like 5 | 0.42 | 1.34 | 0.001 | 0.29 | 1.22 | 0.032 |
| *TLR5* | Toll-like receptor 5 | 0.42 | 1.33 | 0.027 | 0.30 | 1.23 | 0.047 |
| *UBAP2* | Ubiquitin associated protein 2 | 0.41 | 1.33 | 0.030 | 0.27 | 1.20 | 0.347 |
| *SH3BP4L* | SH3 domain-binding protein 4-like | 0.40 | 1.32 | 0.014 | 0.35 | 1.28 | 0.040 |
| *ZHX1* | Zinc fingers and homeoboxes 1 | 0.40 | 1.32 | 0.008 | 0.17 | 1.13 | 0.212 |
| *MIR18B* | Microrna 18b | 0.39 | 1.31 | 0.020 | 0.22 | 1.17 | 0.172 |
| *TSPO* | Translocator protein (18kda) | 0.39 | 1.31 | 0.012 | 0.15 | 1.11 | 0.473 |
| *TOMM6* | Translocase of outer mitochondrial membrane 6 homolog (yeast) | 0.39 | 1.31 | 0.004 | 0.17 | 1.13 | 0.077 |
| *TSKU* | Tsukushi, small leucine rich proteoglycan | 0.39 | 1.31 | 0.041 | 0.32 | 1.25 | 0.201 |
| *LOC415787* | Fatty acyl-coa hydrolase precursor, medium chain-like | 0.39 | 1.31 | 0.009 | 0.34 | 1.26 | 0.029 |
| *TTC38* | Tetratricopeptide repeat domain 38 | 0.39 | 1.31 | 0.029 | 0.11 | 1.08 | 0.563 |
| *MIR1B* | Microrna mir-1b | -0.38 | -1.31 | 0.044 | -0.12 | -1.08 | 0.535 |
| *PKP2* | Plakophilin 2 | -0.38 | -1.31 | 0.022 | -0.09 | -1.06 | 0.513 |
| *EBF3* | Early B-cell factor 3 | -0.39 | -1.31 | 0.005 | -0.29 | -1.22 | 0.069 |
| *WNT2B* | Wingless-type MMTV integration site family, member 2B | -0.39 | -1.31 | 0.011 | -0.12 | -1.09 | 0.180 |
| *THSD7B* | Thrombospondin, type I, domain containing 7B | -0.39 | -1.31 | 0.013 | -0.22 | -1.17 | 0.239 |
| *MIR551B* | Microrna 551b | -0.39 | -1.31 | 0.030 | -0.34 | -1.26 | 0.017 |
| *METTL21EP* | Methyltransferase like 21E, pseudogene | -0.39 | -1.31 | 0.002 | -0.32 | -1.25 | 0.047 |
| *CASC5* | Cancer susceptibility candidate 5 | -0.39 | -1.31 | 0.022 | -0.39 | -1.31 | 0.132 |
| *LOC420039* | Keratin, type I cytoskeletal 42-like | -0.40 | -1.32 | 0.017 | -0.11 | -1.08 | 0.440 |
| *PI16* | Peptidase inhibitor 16 | -0.40 | -1.32 | 0.047 | -0.17 | -1.12 | 0.287 |
| *LRRTM1* | Leucine rich repeat transmembrane neuronal 1 | -0.41 | -1.33 | 0.029 | -0.05 | -1.04 | 0.781 |
| *NSG1* | Neuron specific gene family member 1 | -0.41 | -1.33 | 0.042 | -0.21 | -1.16 | 0.186 |
| *SLC22A2* | Solute carrier family 22 (organic cation transporter), member 2 | -0.42 | -1.34 | 0.002 | -0.14 | -1.10 | 0.101 |
| *NPVF* | Neuropeptide VF precursor | -0.42 | -1.34 | 0.013 | -0.31 | -1.24 | 0.058 |
| *MIR146B* | Microrna 146b | -0.43 | -1.34 | 0.012 | 0.00 | 1.00 | 0.985 |
| *PLK4* | Polo-like kinase 4 | -0.43 | -1.35 | 0.006 | -0.25 | -1.19 | 0.168 |
| *MIR375* | Microrna 375 | -0.43 | -1.35 | 0.007 | -0.16 | -1.11 | 0.305 |
| *SLC25A4* | Solute carrier family 25 (mitochondrial carrier; adenine nucleotide translocator), member 4 | -0.44 | -1.36 | 0.010 | -0.26 | -1.20 | 0.038 |
| *LOC422305* | ES1 protein homolog, mitochondrial-like | -0.44 | -1.36 | 0.012 | -0.24 | -1.18 | 0.064 |
| *TCFL5* | Transcription factor-like 5 (basic helix-loop-helix) | -0.44 | -1.36 | 0.008 | -0.47 | -1.39 | 0.002 |
| *MIR1621* | Microrna mir-1621 | -0.45 | -1.36 | 0.024 | -0.19 | -1.14 | 0.256 |
| *NAE1* | NEDD8 activating enzyme E1 subunit 1 | -0.45 | -1.37 | 0.016 | -0.11 | -1.08 | 0.426 |
| *CHST8* | Carbohydrate (N-acetylgalactosamine 4-0) sulfotransferase 8 | -0.47 | -1.38 | 0.016 | -0.07 | -1.05 | 0.764 |
| *MYH1B* | Myosin, heavy chain 1B, skeletal muscle (similar to human myosin, heavy chain 1, skeletal muscle, adult) | -0.47 | -1.38 | 0.031 | 0.09 | 1.07 | 0.526 |
| *SPRY4* | Sprouty homolog 4 (Drosophila) | -0.49 | -1.41 | 0.020 | -0.35 | -1.27 | 0.066 |
| *MIR302C* | Microrna 302c | -0.50 | -1.41 | 0.031 | -0.29 | -1.22 | 0.090 |
| *RHCE* | Rhesus blood group, ccee antigens | -0.50 | -1.42 | 0.030 | -0.47 | -1.38 | 0.037 |
| *LRRC7* | Leucine rich repeat containing 7 | -0.51 | -1.42 | 0.013 | -0.10 | -1.07 | 0.407 |
| *PRSS35* | Protease, serine, 35 | -0.51 | -1.42 | 0.029 | -0.16 | -1.12 | 0.358 |
| *CYP2W1* | Cytochrome P450, family 2, subfamily W, polypeptide 1 | -0.57 | -1.48 | 0.008 | -0.22 | -1.16 | 0.263 |
| *LOC770434* | Feather keratin Cos1-1/Cos1-3/Cos2-1-like | -0.59 | -1.50 | 0.015 | -0.38 | -1.30 | 0.171 |
| *MOGAT1* | Monoacylglycerol O-acyltransferase 1 | -0.68 | -1.61 | 0.043 | -0.22 | -1.17 | 0.484 |
| *MIR199A2* | Microrna 199a-2 | -0.71 | -1.63 | 0.021 | -0.61 | -1.53 | 0.041 |
| *PCK1* | Phosphoenolpyruvate carboxykinase 1 (soluble) | -1.05 | -2.07 | 0.049 | -0.70 | -1.62 | 0.128 |

The fold changes (FC) were calculated from the signal log ratios (SLR), which were calculated from *n* = 6 microarrays/group.

**Supplemental Table S7** Differentially expressed transcripts (filter criteria: FC > 1.3 and < -1.3 and *P* < 0.05) in the liver of broilers between groups PSA-F25 vs. PSA-F0

| Gene symbol | Gene description | PSA-F25 vs. PSA-F0 | | |
| --- | --- | --- | --- | --- |
|  |  | SLR | FC | *P*-value |
| *MIR1594* | Microrna mir-1594 | 1.41 | 2.66 | 0.018 |
| *GRIN2A* | Glutamate receptor, ionotropic, N-methyl D-aspartate 2A | 1.26 | 2.39 | 0.019 |
| *IP6K2* | Inositol hexakisphosphate kinase 2 | 0.82 | 1.76 | 0.002 |
| *MIR100* | Microrna 100 | 0.72 | 1.65 | 0.021 |
| *ROBO1* | Roundabout, axon guidance receptor, homolog 1 (Drosophila) | 0.68 | 1.61 | 0.001 |
| *RHOB* | Ras homolog family member B | 0.68 | 1.60 | 0.043 |
| *C8ORF22* | Chromosome 2 open reading frame, human c8orf22 | 0.64 | 1.55 | 0.012 |
| *DHRS3* | Dehydrogenase/reductase (SDR family) member 3 | 0.62 | 1.53 | 0.008 |
| *GCNT4* | Glucosaminyl (N-acetyl) transferase 4, core 2 | 0.56 | 1.48 | 0.014 |
| *TMEM86A* | Transmembrane protein 86A | 0.54 | 1.46 | 0.031 |
| *SLC20A2* | Solute carrier family 20 (phosphate transporter), member 2 | 0.52 | 1.44 | 0.036 |
| *FAXDC2* | Fatty acid hydroxylase domain containing 2 | 0.50 | 1.42 | 0.005 |
| *CYP39A1* | Cytochrome P450, family 39, subfamily A, polypeptide 1 | 0.49 | 1.41 | 0.047 |
| *ABCG5* | ATP-binding cassette, sub-family G (WHITE), member 5 | 0.49 | 1.40 | 0.045 |
| *ABCG2* | ATP-binding cassette, sub-family G (WHITE), member 2 (Junior blood group) | 0.48 | 1.40 | 0.046 |
| *CCDC176* | Coiled-coil domain containing 176 | 0.48 | 1.39 | 0.018 |
| *PCMTD1* | Protein-L-isoaspartate (D-aspartate) O-methyltransferase domain containing 1 | 0.47 | 1.38 | 0.024 |
| *NXPH1* | Neurexophilin 1 | 0.45 | 1.37 | 0.005 |
| *TCRA* | T-cell receptor V alpha | 0.44 | 1.36 | 0.000 |
| *MIR29B2* | Microrna 29b-2 | 0.43 | 1.35 | 0.026 |
| *CLYBL* | Citrate lyase beta like | 0.42 | 1.33 | 0.046 |
| *PCDH9* | Protocadherin 9 | 0.42 | 1.33 | 0.033 |
| *CCDC82* | Coiled-coil domain containing 82 | 0.41 | 1.33 | 0.012 |
| *LMO3* | LIM domain only 3 (rhombotin-like 2) | 0.41 | 1.33 | 0.022 |
| *SLC22A4* | Solute carrier family 22 (organic cation transporter), member 4 | 0.41 | 1.32 | 0.015 |
| *RBM46* | RNA binding motif protein 46 | 0.40 | 1.32 | 0.017 |
| *LPIN1* | Lipin 1 | 0.40 | 1.32 | 0.023 |
| *PKD2L1* | Polycystic kidney disease 2-like 1 | 0.40 | 1.32 | 0.026 |
| *SUCLA2* | Succinate-coa ligase, ADP-forming, beta subunit | 0.40 | 1.32 | 0.032 |
| *KIAA1958* | Kiaa1958 | 0.39 | 1.31 | 0.009 |
| *NOX3* | NADPH oxidase 3 | -0.39 | -1.31 | 0.018 |
| *CEP162* | Centrosomal protein 162kda | -0.40 | -1.32 | 0.028 |
| *RNF212* | Ring finger protein 212 | -0.40 | -1.32 | 0.023 |
| *RALGPS2* | Ral GEF with PH domain and SH3 binding motif 2 | -0.41 | -1.33 | 0.032 |
| *RIPK3* | Receptor-interacting serine-threonine kinase 3 | -0.42 | -1.33 | 0.010 |
| *NEXN* | Nexilin (F actin binding protein) | -0.42 | -1.34 | 0.030 |
| *PLAC9* | Placenta-specific 9 | -0.43 | -1.35 | 0.037 |
| *SLC26A2* | Solute carrier family 26 (anion exchanger), member 2 | -0.43 | -1.35 | 0.018 |
| *MIR103-2* | Microrna mir-103-2 | -0.46 | -1.38 | 0.028 |
| *RHCE* | Rhesus blood group, ccee antigens | -0.47 | -1.38 | 0.037 |
| *TCFL5* | Transcription factor-like 5 (basic helix-loop-helix) | -0.47 | -1.39 | 0.002 |
| *BDKRB1* | Bradykinin receptor B1 | -0.49 | -1.40 | 0.016 |
| *ACTA2* | Actin, alpha 2, smooth muscle, aorta | -0.52 | -1.43 | 0.012 |
| *RGS8* | Regulator of G-protein signaling 8 | -0.57 | -1.49 | 0.027 |
| *MIR199A2* | Microrna 199a-2 | -0.61 | -1.53 | 0.041 |
| *NID1* | Nidogen 1 | -0.67 | -1.59 | 0.011 |
| *BG2* | Intestinal zipper protein | -1.63 | -3.10 | 0.040 |

The fold changes (FC) were calculated from the signal log ratios (SLR), which were calculated from *n* = 6 microarrays/group.

**Supplemental Table S8** Validation of selected microarray data by qPCR

| Gene symbol | Fold change | | *P*-value | |
| --- | --- | --- | --- | --- |
|  | Microarray | qPCR | Microarray | qPCR |
| *ABCG2* | 1.49 | 1.56 | 0.010 | 0.012 |
| *COQ10B* | 1.64 | 1.79 | 0.005 | 0.402 |
| *CRYBG3* | 1.26 | 1.02 | 0.003 | 0.917 |
| *CYP2W1* | -1.48 | -1.76 | 0.008 | 0.023 |
| *GPR146* | 1.23 | 1.06 | < 0.001 | 0.744 |
| *ICK* | 1.35 | 1.44 | 0.010 | 0.056 |
| *LRRC7* | -1.42 | -1.38 | 0.013 | 0.248 |
| *MOGAT1* | -1.61 | -1.18 | 0.043 | 0.510 |
| *PDK4* | 1.56 | 1.64 | 0.028 | 0.312 |
| *PLK4* | -1.35 | -1.08 | 0.006 | 0.794 |
| *PRSS35* | -1.42 | -1.18 | 0.029 | 0.604 |
| *RBPMS2* | -1.21 | -1.30 | 0.001 | 0.109 |
| *RHCE* | -1.42 | -1.14 | 0.030 | 0.806 |
| *RUNX2* | -1.22 | -1.01 | 0.001 | 0.954 |
| *TCFL5* | -1.36 | -1.21 | 0.008 | 0.361 |
| *TOMM6* | 1.31 | 1.21 | 0.004 | 0.221 |
| *YPEL5* | 1.34 | 1.06 | 0.001 | 0.600 |
| *ZARL1* | -1.30 | -1.26 | <0.001 | 0.918 |

Data are means for *n* = 6/group (microarray) and *n* = 12/group (qPCR).

**Supplemental Table S9** The most enriched GO terms assigned to the up- and downregulated genes in the liver of broilers of groups PSA-F50 vs. PSA-F0

| GO term | GO category | Count | *P*-value |
| --- | --- | --- | --- |
| *Upregulated transcripts* |  |  |  |
| Potassium channel regulator activity | MF | 3 | 0.001 |
| Voltage-gated potassium channel complex | CC | 3 | 0.002 |
| Potassium channel complex | CC | 3 | 0.002 |
| Transmembrane transporter complex | CC | 4 | 0.004 |
| Extracellular space | CC | 6 | 0.005 |
| Transporter complex | CC | 4 | 0.005 |
| Ion channel regulator activity | MF | 3 | 0.005 |
| Transporter regulator activity | MF | 3 | 0.006 |
| Channel regulator activity | MF | 3 | 0.006 |
| Potassium channel activator activity | MF | 2 | 0.009 |
| Channel activator activity | MF | 2 | 0.009 |
| Transporter activator activity | MF | 2 | 0.009 |
| Plasma membrane protein complex | CC | 4 | 0.011 |
| Cation channel complex | CC | 3 | 0.011 |
| Membrane | CC | 15 | 0.015 |
| Membrane protein complex | CC | 5 | 0.018 |
| Extracellular region | CC | 6 | 0.023 |
| Monoatomic ion channel complex | CC | 3 | 0.032 |
| Molecular function regulator activity | MF | 6 | 0.044 |
| Extracellular matrix | CC | 3 | 0.046 |
| External encapsulating structure | CC | 3 | 0.047 |
| Transmembrane transporter activity | MF | 5 | 0.048 |
| *Downregulated transcripts* |  |  |  |
| Protein localization to plasma membrane | BP | 3 | 0.006 |
| Protein localization to cell periphery | BP | 3 | 0.008 |
| Glycerol metabolic process | BP | 2 | 0.012 |
| Neurotransmitter receptor transport, endosome to postsynaptic membrane | BP | 2 | 0.014 |
| Neurotransmitter receptor transport, endosome to plasma membrane | BP | 2 | 0.014 |
| Alditol metabolic process | BP | 2 | 0.014 |
| Endosome to plasma membrane protein transport | BP | 2 | 0.015 |
| Triglyceride biosynthetic process | BP | 2 | 0.016 |
| Neutral lipid biosynthetic process | BP | 2 | 0.017 |
| Acylglycerol biosynthetic process | BP | 2 | 0.017 |
| Regulation of fibroblast growth factor receptor signaling pathway | BP | 2 | 0.019 |
| Postsynaptic membrane | CC | 3 | 0.019 |
| Neurotransmitter receptor transport to plasma membrane | BP | 2 | 0.020 |
| Establishment of protein localization to postsynaptic membrane | BP | 2 | 0.020 |
| Neurotransmitter receptor transport to postsynaptic membrane | BP | 2 | 0.020 |
| Protein localization to membrane | BP | 3 | 0.027 |
| Neurotransmitter receptor transport | BP | 2 | 0.027 |
| Protein localization to postsynaptic membrane | BP | 2 | 0.027 |
| Protein localization to postsynapse | BP | 2 | 0.027 |
| Synaptic membrane | CC | 3 | 0.031 |
| Establishment of protein localization to plasma membrane | BP | 2 | 0.032 |
| Protein localization to synapse | BP | 2 | 0.032 |
| Triglyceride metabolic process | BP | 2 | 0.037 |
| Regulation of postsynaptic membrane neurotransmitter receptor levels | BP | 2 | 0.037 |
| Localization within membrane | BP | 3 | 0.038 |
| Receptor localization to synapse | BP | 2 | 0.038 |
| Protein localization to cell junction | BP | 2 | 0.044 |
| Carbohydrate metabolic process | BP | 3 | 0.047 |
| Regulation of biological quality | BP | 5 | 0.048 |

*GO terms are sorted by their enrichment *P*-value in increasing order. Only GO terms with enrichment *P*-value < 0.05 are shown. Abbreviations: GO, gene ontology; BP, biological process; CC, cellular component; MF, molecular function.
